# Early and non-destructive prediction of the differentiation efficiency of human induced pluripotent stem cells using imaging and machine learning

**DOI:** 10.1038/s41598-025-11108-5

**Published:** 2025-07-23

**Authors:** Miki Arai Hojo, Taku Tsuzuki, Yosuke Ozawa, Toshiyuki Araki, Hidetoshi Sakurai

**Affiliations:** 1 Department of Clinical Application, Center for iPS Cell Research and Application (CiRA), Kyoto University, Kyoto, Japan; 2 Epistra Inc, Tokyo, Japan; 3 Department of Peripheral Nervous System Research, National Institute of Neuroscience, National Center of Neurology and Psychiatry (NCNP), Tokyo, Japan

**Keywords:** Human induced pluripotent stem cells (hiPSCs), Early and non-destructive prediction, Bioimage informatics, Directed differentiation, Muscle stem cells (MuSCs), Machine learning, Pluripotent stem cells, Image processing, Machine learning

## Abstract

The reproducibility and robustness of many directed differentiation protocols from human induced pluripotent stem cells (hiPSCs) remain low, and the long differentiation induction period significantly limits protocol optimization. To address this, we developed an early and non-destructive prediction system for the differentiation induction efficiency of hiPSCs using bioimage informatics. We employed a directed differentiation protocol for muscle stem cells (MuSCs), a promising cell source for the regenerative therapy of muscular dystrophy. Biological analyses suggested that days 14–38 are positive for forecasting the induction efficiency on day 82. Therefore, we conducted six independent experiments, inducing MuSC differentiation in a total of 34 wells, and captured a total of 5,712 phase contrast cell images between days 14 and 38. We selected Fast Fourier transform (FFT) as the feature extraction method and confirmed that it captures the characteristics of cells during differentiation. By classifying images on each day using extracted features and machine learning, we found that samples with high and low induction efficiency could be predicted at approximately 50 days before the end of induction. This system is expected to contribute to regenerative therapy through effective protocol optimization.

## Introduction

Human induced pluripotent stem cells (hiPSCs) are generated from adult somatic cells, and have infinite proliferation ability as well as the ability to differentiate into cells from any of the three germ layers^1,2^. Consequently, hiPSCs can be applied in a number of applications, such as regenerative therapy, drug discovery screening, disease modeling, and human developmental biology^2–6^. Currently, two strategies are used to support the differentiation of hiPSCs into specific somatic cells, namely direct reprogramming and directed differentiation^7^. In direct reprogramming, differentiated cells can be stably obtained in a short period via the forced expression of genes such as master transcription factors^7–9^. However, direct reprogramming is fundamentally unsuitable for clinical applications due to several limitations, including the risk of tumorigenesis, the generation of off-target cells, and the production of differentiated cells with extremely low maturation states^7,10,11^. Directed differentiation is a transgene-free method whereby cells are differentiated by culturing in a medium containing growth factors, differentiation factors, and small molecules, thus mimicking developmental processes^5,10,12–21^. This method carries a lower risk of tumorigenesis and often produces more mature differentiated cells compared to direct reprogramming methods^10^. Therefore, it is desirable to use cells and tissues differentiated via the directed differentiation method for regenerative therapy.

It is well known that the reproducibility and robustness of many directed differentiation protocols remain low. The induction efficiency varies in hiPSC clones, experimental batches, and even in the wells of plates due to the variability of the genetic and epigenetic state in each hiPSC clone^4,22–28^, the researcher’s skill^29,30^, slight changes in seeding cell number, and bias of seeded cells in a culture dish. In addition, directed differentiation protocols for some cell types take several months for induction^5,10,16,17,20,21,31^. At present, there are very few methods that can be used to select samples with high induction efficiency at an early stage. This makes it difficult to elucidate important factors for improving and stabilizing induction efficiency. Moreover, assessing the properties of differentiated cells is laborious.

Therefore, novel methods are necessary to predict final differentiation induction efficiency at early time points. Although the assays to measure the expression of marker genes or proteins are currently common for evaluating cell conditions^10,13,32,33^, they require skilled techniques, are time-consuming, costly, and most importantly, destructive to cells. Appropriate prediction methods must be non-destructive because the induction efficiency often varies in wells, even in the same experimental batch and condition. Phase contrast imaging is one of the most simple, low-cost, and non-destructive methods used to observe cell conditions. Recently, many studies have succeeded in estimating cell states from phase contrast cell images by combining imaging with machine learning^34–42^. Some examples include; detecting physical behavior of cells^34^, determining whether cells are disease-related or not, determining the quality of differentiation, genetic interactions, gene and protein expression levels, and quantifying the number of days from the start of differentiation^35–42^. One limitation of these studies is that the properties of cells are judged at the time when the images are taken and these studies have not been able to predict the future cell condition. With regard to mesenchymal stem cells (MSCs) and hematopoietic stem cells (HSCs), some studies have succeeded in forecasting future differentiation states by utilizing computational image analysis or deep learning^43–45^. However, the number of cell lineages that can differentiate from MSCs and HSCs is significantly less than that from iPSCs. Furthermore, the differentiation induction period in these studies was up to 25 days, and predictions were performed 18 hours to 7 days prior to the final day of differentiation. Some directed differentiation protocols from hiPSCs may even take several months for induction^5,10,16,17,20,21^, which is currently a major bottleneck for the improvement of the reproducibility and robustness of these protocols. Therefore, to promote regenerative therapy, it is crucial to establish a method that is able to predict the differentiation state of hiPSCs at a very early stage using simple, low-cost, and non-destructive techniques, such as imaging.

**Fig. 1:**
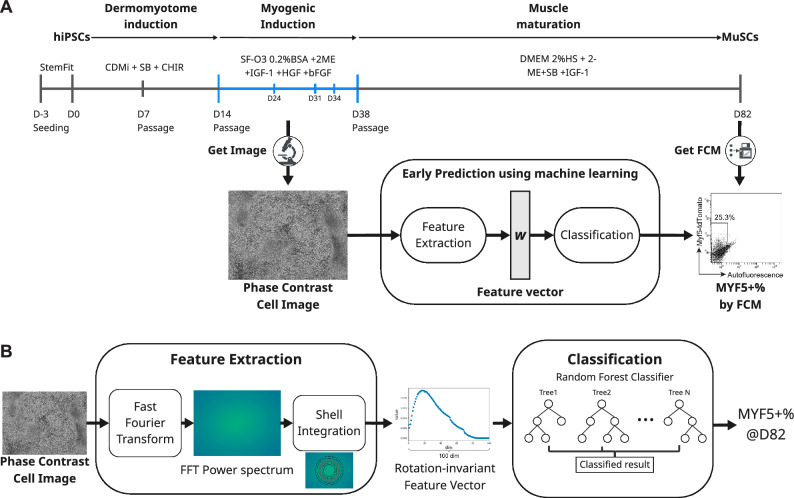
Schematic overview of the experimental workflow and the machine learning system for early prediction of muscle stem cell (MuSC) differentiation efficiency (**A**) Schematic representation of the directed differentiation protocol for MuSCs and the general concept of the early prediction system using machine learning, which takes phase contrast images as input, performs feature extraction and classification to predict the final MYF5 + % (day 82). (**B**) Detailed workflow of the early prediction system. The Feature Extraction process begins with a phase contrast cell image. A Fast Fourier Transform (FFT) is applied to the image to obtain an FFT power spectrum. Subsequently, shell integration is performed on the power spectrum to generate a rotation-invariant feature vector, designed to capture morphological characteristics of the cells. This feature vector then serves as input for the Classification process. A Random Forest Classifier utilizes the extracted feature vector to predict the MYF5 + % (day 82).

In this study, we developed a system comprising phase contrast imaging and machine learning that is able to predict the final differentiation induction efficiency approximately 50 days prior to the induction period, which is less than half of the total induction period. We employed a directed differentiation protocol for the induction of muscle stem cells (MuSCs) from hiPSCs^10,46^ that was developed by using muscle satellite cell marker MYF5-tdTomato reporter hiPSCs^10^. The previous report revealed that MYF5 + MuSCs have regenerative capacity for damaged muscle in Duchenne muscular dystrophy (DMD) model mice^10^; therefore, they are believed to be promising cell sources for regenerative therapy for muscular dystrophy. However, MuSC induction is a long-term process (approximately 80 days), with low reproducibility and robustness. By investigating gene and protein expression, we clarified that the expression of myogenic induction markers on day 38 were correlated with MuSC induction efficiency on day 82. From these results, we hypothesized that the final induction efficiency is predictable through cell imaging during myogenic induction phase from day 14 to 38. To test this hypothesis, and building upon observations of images acquired from day 14 to 38, we designed and validated a machine learning-based system to predict MuSC induction efficiency on day 82 (Fig. 1). Our system is comprised of two main processes: a Fast Fourier Transform (FFT)-based feature extraction process, followed by a classification process. In the feature extraction process, FFT is applied to each acquired phase contrast cell image to obtain its power spectrum. Subsequently, shell integration is performed on this power spectrum to generate a 100-dimensional, rotation-invariant feature vector, which is designed to capture morphological characteristics of the cells. In the subsequent classification process, these extracted feature vectors are utilized by a random forest classifier to predict the MuSC induction efficiency on day 82 (Fig. 1). Our findings demonstrate that this system enables highly accurate prediction of samples with both high and low MuSC induction efficiency. Specifically, predictions made using images from days 31 or 34 for high-efficiency samples, and day 24 for low-efficiency samples, were particularly effective. In fact, classification using images from day 24 and day 34 resulted in a 43.7% reduction in the defective sample rate and a 72% increase in the number of good samples.

## Results

### The myogenic induction phase can be used to predict MuSC induction efficiency on day 82

In our MuSC induction protocol, dermomyotome cells were induced from hiPSCs via treatment with a Wnt agonist at a high concentration for 14 days (Fig. 1). Next, the dermomyotome cells were treated with three types of growth factors; insulin-like growth factor 1 (IGF-1), hepatocyte growth factor (HGF), and basic fibroblast growth factor (bFGF) for 3 weeks in order to promote myogenic differentiation (Fig. 1). The induced myotubes were then matured by switching the culture medium to a conventional muscle culture medium based on a low concentration of horse serum (Fig. 1). MuSCs were obtained around day 80 (Fig. 1).

**Fig. 2:**
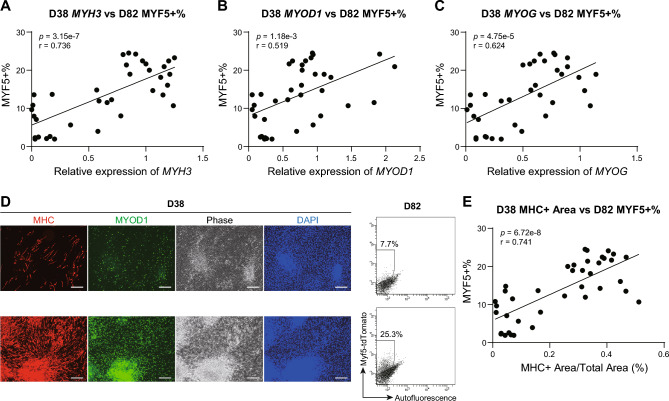
The myogenic induction phase can be used to predict MuSC induction efficiency on day 82 (**A-C**) Correlation analysis between the MYF5 + % score on day 82 and relative mRNA expression of *MYH3* (**A**), *MYOD1* (**B**), or *MYOG* (**C**) on day 38 (normalized to *HPRT1*). Data are pooled from 36 wells of experiments. The mRNA expression levels were examined using separate wells from those used for MYF5 + % analysis and were cultured using the same experimental conditions. (**D**) The results of immunocytochemistry (ICC) for myosin heavy chain (MHC) and MYOD1 on day 38, and flow cytometry (FCM) analysis for MYF5 + % on day 82. Data are representative of 39 wells of experiments. ICC was performed using separate wells from those used for MYF5 + % analysis and were cultured using the same experimental conditions. The scale bar represents 500 μm. (**E**) Correlation analysis between the MYF5 + % score on day 82 and MHC + area calculated from images of ICC on day 38. Data are pooled from 39 wells of experiments. Between two to five images per well were taken at different positions.

First, we investigated which phase of MuSC induction is suitable for prediction. *MYF5* has been reported to be expressed in satellite cells^47^. Previously, we generated MYF5-tdTomato reporter hiPSCs and induced differentiation into MuSCs using our protocol^10^. The resulting MYF5 + MuSCs expressed PAX7, a well-established marker of satellite cells^7^, as well as other fetal muscle stem cell markers, and further demonstrated to have the ability to repair damaged muscle *in vivo*^10^. Therefore, previously generated MYF5-tdTomato reporter hiPSCs^10^ were used to analyze the final MuSC induction efficiency via flow cytometry (FCM) on day 82. We performed quantitative real-time RT-PCR (qRT-PCR) for the representative genes on days 7, 14, and 38, and investigated the correlations to the MYF5 positive percentage (MYF5 + %) on day 82. On day 7, the expression of an early mesoderm marker, *T*^48^, a paraxial mesoderm marker, *TBX6*^49^, and a dermomyotome marker, *SIX1*^50^ were measured, and we observed no significant correlations to MYF5 + % (Supplementary Fig. 1). On day 14, the expression of dermomyotome markers, *DMRT2*, *PAX3*, and *SIX1*^32,50,51^ were determined, and positive correlations were not observed (Supplementary Fig. 1). In contrast, significant positive correlations were detected between the expression of skeletal muscle markers, *MYH3*^52^, *MYOD1*^53^, and *MYOG*^54^ on day 38 and MYF5 + % on day 82 (Fig. 2). To confirm that these correlations were preserved at a protein level, immunocytochemistry (ICC) was performed. We found that samples with high myosin heavy chain (MHC) and MYOD1 expression tended to exhibit high MYF5 + % on day 82 (Fig. 2). The MHC expressing area was quantified and was found to exhibit a significant positive correlation with MYF5 + % on day 82 (Fig. 2). We also investigated whether MHC expression at day 38 correlated with the final MuSC differentiation induction efficiency in five additional cell lines (Supplementary Fig. 1). The differentiation induction efficiency in these cell lines was assessed using CDH13, a cell surface marker for MuSCs that we previously identified^53^. As a result, a significant positive correlation was observed between the MHC-positive area and the final CDH13 positivity rate (Supplementary Fig. 1). These results suggest that monitoring the myogenic induction phase is promising for the prediction of MYF5 + % on day 82.

### The FFT-based feature extraction process was suitable for the prediction of MuSC induction efficiency using data from the myogenic induction phase

**Fig. 3:**
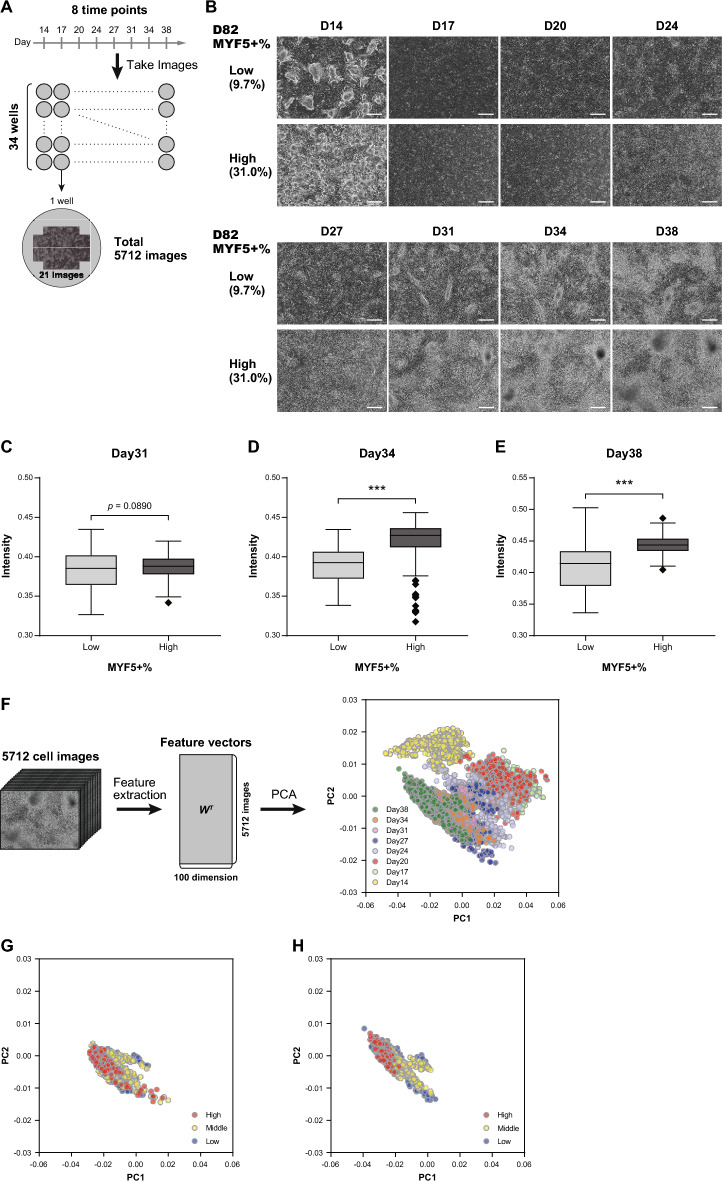
FFT-based feature extraction process was suitable for the prediction of MuSC induction efficiency from the myogenic induction phase. (**A**) Schematic representation of the image acquisition procedure. Images of each of the 34 wells were taken on eight separate occasions using 21 different positions. A total of 5,712 images were obtained. (**B**) Phase contrast cell images from day 14 to 38. Images are representative of 21 different points of view in the well and 34 wells of experiments. The scale bar represents 500 μm. (**C-E**) Comparison of the intensity of the high-frequency components between high and low MYF5 + % score groups on day 31 (**C**), day 34 (**D**), and day 38 (**E**). In each plot, the left bar represents the distribution of a low MYF5 + % score group and the right bar represents the distribution of a high MYF5 + % score group. Box-and-whisker plot is drawn in the style of Tukey. ***p < 0.001. (**F**) Principal component analysis (PCA) for feature vectors from 5,712 images. The left side shows the feature extraction procedure, and the right scatter plot represents the PCA results. In the PCA plot, the horizontal axis represents the first component of PCA (PC1) and the vertical axis represents the second component of PCA (PC2). Each point in the scatter plot represents the value of each image. The colors of each point represent the day on which each image was taken. (**G-H**) PCA results on day 34 (**G**) and day 38 (**H**). The colors of each point represent the MYF5 + % score (high: > 20%, middle: 10–20%, low: < 10%).

Based on the results displayed in Fig. 2, phase contrast cell images obtained on eight separate occasions between day 14 and 38 were used for subsequent experiments (Fig. 3, Supplementary Figs. 2–9). We performed six independent MuSC differentiation induction experiments, with 4–6 technical replicates per experiment, resulting in a total of 34 wells. For each well, 21 images were captured from different positions (Fig. 3, Supplementary Figs. 2–9). In total, 5,712 images were acquired for MYF5 + % prediction (Fig. 3, Supplementary Figs. 2–9). It was previously reported that the maximum MYF5 + % score for this particular differentiation induction protocol was approximately 30^54^. In our experiments, this result was reproduced and the maximum MYF5 + % score was 31. When purifying MuSCs with a cell sorter to evaluate MuSC properties, samples with a MYF5 + % score over 20 can be efficiently collected, whereas samples with a MYF5 + % score under 10 take a long time to sort and cells are often damaged. Therefore, we defined a high and low MYF5 + % score as more than 20 and less than 10, respectively.

Although deep learning has become common in image classification, it requires a substantial number of independent experiments for image collection, and our 34 experiments were insufficient^55^. Therefore, we decided to design a feature extraction process based on our observations of the images. By qualitatively comparing images with high and low MYF5 + % scores, we found that cell density seemed to be higher in the samples with high MYF5 + % scores between day 31 and 38 (Fig. 3, Supplementary Figs. 2–9). Ruggeri et al. previously reported that a circular band in a FFT spectrum of an image contains frequency information related to cell density^56^; therefore, we designed a feature extraction process based on FFT. Our process comprised the following two steps: First, FFT was applied to a cell image to obtain a normalized FFT power spectrum. Then a shell integration was adopted to the normalized FFT power spectrum to obtain a rotation-invariant 100-dimensional feature vector. To objectively confirm our observation that the MYF5 + % score on day 82 correlated with the cell density between day 31 and 38, we compared the intensity of high-frequency components that should reflect a high cell density^56^ using the images with high and low MYF5 + % scores (Fig. 3). The intensity of high-frequency components was defined as a sum of the intensities of components above the 30th feature vector. We investigated the images taken on days 31, 34, and 38 (Fig. 3) and found that the intensity was significantly higher in the group with a high MYF5 + % score on days 34 and 38 (Fig. 3). On day 31, the difference between the high and low MYF5 + % score groups was not significant, but the intensity of the high MYF5 + % score group tended to be higher (Fig. 3). These results suggest that the cell density in the late myogenic induction phase was related to the MYF5 + % score on day 82.

In addition, we obtained feature vectors for each of the 5,712 cell images and visualized them using principal component analysis (PCA) (Fig. 3 and Supplementary Fig. 11). Each cell image was grouped according to the date of differentiation, and the feature vectors for day 14 (only the dermomyotome induction phase) were plotted at a different point when compared to the other vectors of the myogenic induction phase (Fig. 3 and Supplementary Fig. 11). This indicates that the FFT-based feature extraction process could capture the morphological changes that occur with differentiation. When comparing the results of the PCA analysis on day 34 and day 38 based on the MYF5 + % scores (high: > 20%, middle: 10–20%, low: < 10%), the dots in the high group appeared relatively clustered, whereas those in the middle and low groups were more dispersed (Fig. 3 and Supplementary Fig. 11). These results suggest that the feature extraction method have the potential to capture morphological characteristics of cells associated with the MYF5 + % score. Using the results displayed in Fig. 3, we concluded that the use of the FFT-based feature extraction method was viable for the prediction of the MYF5 + % score on day 82.

### High and low MYF5 + % scores were predictable approximately 50 or more days before the end of the inductionperiod

**Fig. 4:**
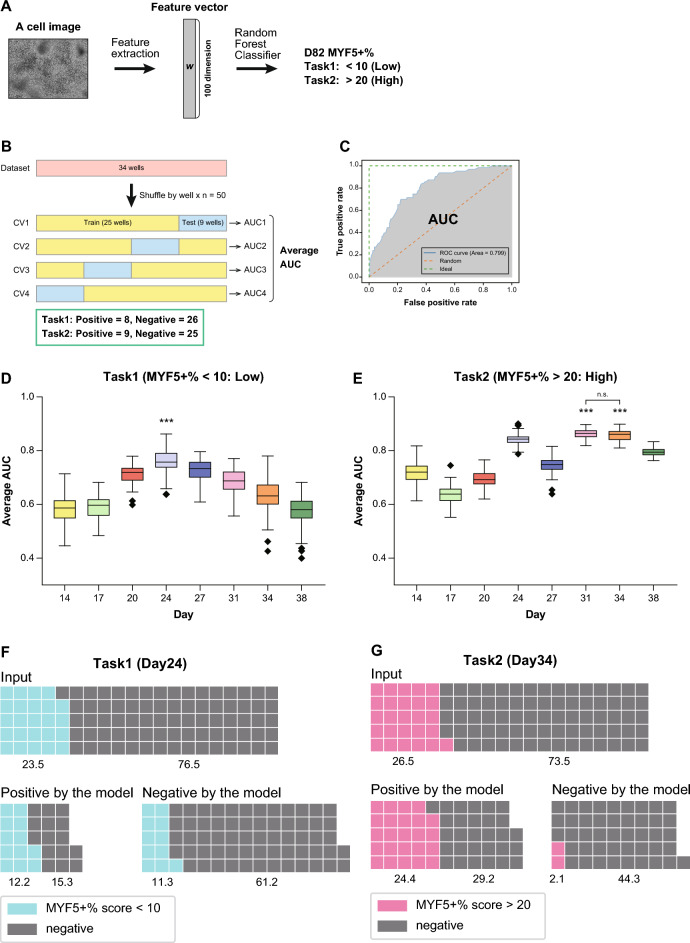
High and low MYF5 + % scores were predictable approximately 50 or more days before the end of the induction period. (**A**) Schematic representation of the image classification procedure. Using the feature vectors extracted from the FFT, images were classified according to whether the MYF5 + % score was less than 10 (Task 1) or more than 20 (Task 2) by a random forest classifier. (**B**) Schematic explanation of the process used for the performance test of the classifier. Thirty-four wells of data were acquired and divided into either training data (25 wells) and test data (9 wells). Four-fold cross-validation (CV) was performed, and the average area under the curve (AUC) was calculated. The same trial was repeated 50 times; however, the combination of each data set was changed for each repeat. These results were used to calculate the distribution of average AUCs. (**C**) Schematic explanation of the AUC. The AUC is the area under the receiver operating characteristic curve (ROC curve). The ROC curve is shown in blue. The AUC corresponds to the grey-filled region. (**D-E**) Performance tests of the classifier for Task 1 (**D**) or Task 2 (**E**). The horizontal axis represents the number of days from the start of differentiation. The vertical axis represents the average AUC value. Data are pooled from each average AUC derived from the 50 performance tests. The box-and-whisker plot is drawn in the style of Tukey. ***q < 0.001 to all other days. n.s. not significant. (**F-G**) Waffle plots illustrating the classifier’s performance when used as a filter in Task 1 (**F**) and Task 2 (**G**). Each plot contains 100 squares, with each square representing 1% of the total cell images, normalized for visual clarity. Colored boxes indicate positive samples for each task (Task 1: MYF5 + % < 10%, Task 2: MYF5 + % > 20%). “Input” shows the initial distribution of samples. “Positive by the model” shows the distribution of samples classified as positive by the classifier. “Negative by the model” shows the distribution of samples classified as negative by the classifier.

In this study, we determined two classification tasks, Task 1 and 2, based on our definition of high and low MYF5 + % scores (Fig. 4). A 100-dimensional feature vector was extracted using the FFT-based process (Figs. 3 and 4). Cell images were classified using the extracted feature vectors and the random forest classifier^57^, and we determined whether they met the requirements of Task 1 or 2 (Fig. 4 and Supplementary Fig. 10).

To determine which days were optimal for predicting high and low MYF5 + % scores, we measured the classifier’s performance using images from each day. For each date, the 34 wells of data were divided into either training data (25 wells) or test data (nine wells), and the classifier was trained using the training data (Fig. 4). The performance of the trained classifier was evaluated using test data and the area under the curve (AUC) was calculated (Fig. 4). The AUC is the area under the receiver operating characteristic curve (ROC curve) and is generally used as an index of the performance of a classifier (Fig. 4)^58^. We performed four-fold cross-validation (CV) and obtained an average AUC (Fig. 4). Furthermore, we performed the same evaluation trial 50 times and changed the combination of train and test data division in each separate trial. Finally, distributions of the average AUCs for each day and Task were acquired (Fig. 4). In Task 1 (Low: MYF5 + % score < 10), the average AUCs on day 24 were significantly higher than those on the other days (median: 0.76), and they decreased as the induction period progressed (Fig. 4). In Task 2 (High: MYF5 + % score > 20), the average AUCs were significantly higher on days 31 and 34 (median: 0.86), followed by day 24 (Fig. 4). We concluded that these specific days are appropriate for the prediction of samples with high and low induction efficiency.

The results displayed in Fig. 4 indicate that features correlating to high and low MuSC induction efficiency were included in the images obtained on days 31 or 34, and 24 respectively. To consider features other than cell density that may affect the performance of the classifier, we monitored the expression of MHC, which is the structural protein of myotubes, using ICC during the myogenic induction phase (days 17–38). We found that MHC expression began around day 24 and was strongly expressed from day 31 (Supplementary Fig. 12). Based on these results, we consider that morphological changes associated with MHC expression may have been one of the features utilized by the classifier in addition to cell density.

Next, we evaluated the performance of the classifier at day 24 for Task 1 and day 34 for Task 2 after applying the classifier as a filter to improve yield in the manufacturing process. The evaluation results are shown in Fig. 4.

For Task 1, which detects and excludes images with MYF5 + % scores below 10 (defective products) as positive, we assessed the performance of the classifier using two metrics: False Positive Rate (FPR) and Impurity Change. The FPR indicates the proportion of images that actually have MYF5 + % scores above 10 but were incorrectly detected as below 10. Here, the FPR was 0.19, indicating that 20% of the images, which were not defective, were erroneously classified as defective. Impurity Change measures the change in the proportion of images with MYF5 + % scores below 10 in the dataset before and after classification. Initially, among the 714 cell images, 147 (20.6%) had MYF5 + % scores below 10. After classification, 196 images were excluded as defective, leaving 518 images. Of these, 60 images (11.6%) had MYF5 + % scores below 10, resulting in an Impurity Change of 0.116/0.206 = 0.563. This significantly reduced the defect rate by 43.7% (p = 2.91 × 10^−20^) (Fig. 4).

For Task 2, which detects and retains images with MYF5 + % scores above 20 (high-quality products) as positive, we evaluated the classifier’s performance using False Negative Rate (FNR) and Impurity Change. The FNR indicates the proportion of images that actually have MYF5 + % scores above 20 but were incorrectly detected as below 20. Here, the FNR was 0.078, indicating that 7.8% of the high-quality images were mistakenly classified as lower quality. Impurity Change measures the change in the proportion of images with MYF5 + % scores above 20 in the dataset before and after classification. Initially, among the 714 cell images, 189 (26.5%) had MYF5 + % scores above 20. After classification, 383 images were retained as high-quality. Of these, 174 images (45.5%) had MYF5 + % scores above 20, resulting in an Impurity Change of 0.455/0.265 = 1.719. This significantly concentrated the high-quality product rate by 72% (p = 1.10 × 10^−39^) (Fig. 4).

Based on these results, we concluded that the classifiers we constructed for Task 1 and Task 2 were useful for application scenarios. In particular, classifier for Task 2 demonstrated high effectiveness.

## Discussion

Directed differentiation from hiPSCs is suitable for regenerative therapy because it has a low risk of tumorigenicity. Currently, there are no available methods that can be used to predict final differentiation induction efficiency at an early stage, and at present, many protocols take several months for induction, with low reproducibility and robustness. In this study, we succeeded in predicting the future differentiation induction efficiency using phase contrast cell images in the early phase of differentiation by designing an image classification process with FFT and a random forest classifier based on biological investigation and visual image comparison. We also demonstrated the performance of our classifier, showing that it is particularly effective in enriching samples that exhibit high differentiation efficiency.

Our directed differentiation protocol for MuSCs consisted of three phases and took approximately 80 days to complete (Fig. 1). First, to investigate which phase could be used to predict the final induction efficiency, we analyzed the expression of representative genes in each phase and determined their correlation to the final differentiation induction efficiency (Fig. 2 and Supplementary Fig. 1). We found significant positive correlations between the expression of myogenic-related genes and proteins on day 38 and the MYF5 + % score on day 82 (Fig. 2). These results suggest the following: First, in our protocol, MuSCs were induced with other muscle lineage cells^57^. Therefore, the samples with a high myogenic induction efficiency on day 38 were considered to have high differentiation efficiency of whole myogenic-lineage cells, including MuSCs. Second, the MuSCs induced using our protocol exhibited a fetal muscle stem cell-like phenotype^58^, and a previous study has reported the importance of myofibers as a niche of muscle stem cells *in vivo*^59^. Therefore, it is presumed that the high degree of differentiation of myotubes on day 38 promoted the differentiation of the MuSCs. To improve the robustness of the differentiation induction of MuSC, it could be crucial to optimize the protocol of the myogenic induction phase.

Based on our qualitative observation, we developed a process that extracts image features using a FFT and predicts high and low MYF5 + % scores by processing the features via a random forest classifier (Figs. 3 and 4). Surprisingly, it was revealed that we could predict what the high and low MYF5 + % scores would be on day 82 with high accuracy on days 31 or 34, and 24, respectively, which is almost 50 days prior to the end of the differentiation induction period (Fig. 4). Furthermore, it was shown that images on day 24 and day 34 contributed to the elimination of bad samples and the selection of good samples (Fig. 4). Until now, many studies have reported estimate cell states, such as the gene expression level, using cell imaging combined with machine learning^35–42^. However, in these reports, the cell states were judged only at the time of the image being taken. Some studies on the differentiation of MSCs and HSCs have forecasted future differentiation states using cell images. Kazemimoghadam et al. identified a suitable prediction point that can be used to judge high neural differentiation potential using computational image analysis^5,10,16,17,20,21^. Buggenthin et al. and Matsuoka et al. successfully predicted future hematopoietic lineage choice and osteogenic differentiation potential using cell imaging combined with deep learning^54^. However, the induction periods used in these studies were all shorter than 1 month, which is substantially shorter than many directed differentiation protocols for hiPSCs^5,10,16,17,20,21^. Furthermore, prediction points were only obtained between 18 hours and 7 days before the end of the induction period. In contrast, in the present study, we succeeded in predicting the differentiation induction efficiency after 82 days of culture using images taken approximately 50 days prior, which was during the early phase of differentiation induction. This could contribute significantly to the improvement of reproducibility and robustness in differentiation induction. In addition, iPSCs have greater pluripotency than MSCs and HSCs, and this study demonstrated the feasibility of cell image-based future prediction for differentiated cells derived from hiPSCs. It is important to develop early-stage prediction methods for other hiPSC-derived differentiation induction systems in order to widely promote regenerative therapy.

A previous study conducted by Ruggeri et al. demonstrated that FFT can be used to obtain information on cell density^56^. Figure 3 demonstrated that the high-frequency component was significantly higher in the high MYF5 + % score group on days 38 and 34, whereas no significant difference was observed on day 31 (Fig. 3). However, the accuracy for the prediction of both high and low MYF5 + % scores was substantially higher on day 31 than on day 38 (Fig. 4). Furthermore, it was clarified that images on day 24 were more suitable for the prediction of low MYF5 + % scores (Fig. 4) even though no big difference in cell density was observed on day 24 (Fig. 3 and Supplementary Fig. 5). These results suggest that, in addition to cell density-related features, the random forest classifier used some morphological characteristics for image classification. In Supplementary Fig. 12, we indicated that the day on which MHC expression was largely increased corresponded with the day with high prediction accuracy (Supplementary Fig. 12). Therefore, it is suggested that morphological changes related to MHC expression were one of the features used for prediction.

By adopting our proposed method, we succeeded in constructing a classifier that can accurately predict the final MYF5 + % scores (approximately 0.8 AUC) (Fig. 4) and concentrate samples with high differentiation efficiency (Fig. 4). However, some samples were not classified correctly by our classifier. One possible approach that can be used to improve the classifier’s performance is to increase the number of experiments conducted to obtain samples for training. As more data is accumulated from experiments, we expect to improve the classifier’s performance by extracting more informative features from images using more flexible methods such as deep learning.

In this study, we demonstrated the potential to eliminate samples with failed differentiation and enrich those with successful differentiation around days 24–34. Although the culture period has been significantly reduced compared to the conventional ~ 80 days, achieving even earlier prediction would be more desirable from the perspective of minimizing cell culture labor and expenses. In image analysis, we used the images on day 14 as the control because day 14 fell within the dermomyotome induction phase (Fig. 2), and the expression of representative genes on day 14 were not correlated with the MYF5 + % score on day 82 (Supplementary Fig. 1). Nevertheless, the average AUCs for the prediction of a high MYF5 + % score on day 14 were relatively high (approximately 0.7 AUC) (Fig. 4). This suggests that some important factors for MuSC induction could be detected even on day 14 if we developed a higher-resolution image analysis method or another non-destructive method. In conclusion, we developed a system that is able to predict and select the samples with high and low induction efficiency of MuSCs using phase contrast cell images. Conventionally, the determination of the cell condition in the differentiation process has required an expert’s eye. Using this system, we can determine the final induction efficiency with consistency approximately 50 days before the induction period is complete, which is less than half of the total induction period. This could contribute to elucidating important factors that can be used to improve the reproducibility and robustness of differentiation induction; thus, reducing the cost and time for experiments. Furthermore, this is expected to promote the evaluation and leverage of differentiated cells through their effective production, and we believe that this system may accelerate hiPSC-based regenerative therapy. As a next step, we should focus on testing whether a similar method could be applicable to another directed differentiation protocol.

## Methods

### hiPSC line and maintenance culture

The hiPSC clone 201B7-derived^58^ MYF5-tdTomato reporter line was used for MuSC differentiation induction. The original 201B7 cell line distributed by RIKEN BRC has been confirmed to have a normal karyotype through karyotypic analysis (https://cellbank.brc.riken.jp/cell_bank/CellInfo/?cellNo=HPS0063&lang=En). The MYF5-tdTomato reporter line was previously established by a CRISPR-Cas9-based knock-in system^59^. hiPSCs were cultured and maintained under feeder-free culture conditions as previously described^60^. Briefly, cells were passaged at 2,000–10,000 cells/well in 6-well plates coated with iMatrix-511 (Nippi, Incorporated, Japan) once a week and maintained in 2 mL/well of StemFit AK02 medium (Ajinomoto, Japan). The medium was replaced three times per week with fresh StemFit AK02 medium.

For Supplementary Fig. 1, we used five patient-derived iPSC clones: A clone derived from a Duchenne muscular dystrophy (DMD) patient (Clone ID: CiRA00111, which was referred to as DMDΔ44-1 in our previous report^60^), a clone derived from a myotonic dystrophy type 1 (DM1) patient (Clone ID: CiRA00211, which was referred to as Patient-2 in our previous report^61^), a clone derived from a facioscapulohumeral muscular dystrophy (FSHD) type 1 patient (Clone ID: CiRA00655, which was referred to as F1#1 in our previous report^62^), a clone derived from a laminopathy patient (Clone ID: CiRA00457, which was referred to as LMNA in our previous report^63^), and a clone derived from a congenital myasthenic syndrome (CMS) patient who had a mutation in *GFPT1* gene (c.722–723insG) (Clone ID: M16#66). All patient-derived iPSC clones were established by overexpression of Oct3/4, Sox2, L-Myc, Klf4, and Lin28 by using episomal vectors.

### Directed differentiation of MuSCs from hiPSCs

Differentiation induction of MuSCs was performed following our previously reported protocol^63^. hiPSCs were dissociated with Accutase (Nacalai tesque, Japan) and plated at 5,000–20,000 cells/well on Matrigel (Corning, NY, USA)-coated 6-well plates in 2 mL/well of StemFit AK02 medium supplemented with 10 μM Y-27632 (Nacalai tesque). After 2 days, the medium was replaced with StemFit AK02. On the following day, the medium was switched to a chemically defined medium (CDMi) supplemented with 5 μM SB431542 (SB, Nacalai tesque or FUJIFILM Wako Pure Chemical Corporation, Japan) and 10 μM CHIR99021 (CHIR, FUJIFILM Wako Pure Chemical Corporation), and dermomyotome induction was initiated (day 0). CDMi is composed of Iscove’s Modified Dulbecco’s Medium (IMDM, FUJIFILM Wako Pure Chemical Corporation, 098–06,465) and Ham’s F-12 (FUJIFILM Wako Pure Chemical Corporation, 087–08,335) at a 1:1 ratio and is supplemented with 1% bovine serum albumin (BSA, Sigma-Aldrich, STL, USA, A9418), 1% Penicillin–Streptomycin Mixed Solution (Nacalai tesque), 1% CD Lipid Concentrate (Thermo Fisher Scientific, Inc., MA, USA), 1% Insulin-Transferrin-Selenium (Thermo Fisher Scientific, Inc.), and 450 μM 1-Thioglycerol (Sigma-Aldrich). On days 3 and 5, the medium was replaced with the same medium that was used on day 0. On day 7, cells were dissociated with Accutase and plated at 300,000–450,000 cells/well on Matrigel-coated 6-well plates in 2 mL/well of CDMi supplemented with 5 μM SB, 10 μM CHIR, and 10 μM Y-27632. The medium was changed on days 10 and 12. On day 14, cells were dissociated again with Accutase and seeded at 400,000–800,000 cells/well on Matrigel-coated 6-well plates in 2 mL/well of CDMi supplemented with only 10 μM Y-27632. Three days later (day 17), the medium was switched to S-clone SF-O3 (SF-O3, Sekisui Medical Company, Limited, Japan) supplemented with 0.2–0.4% BSA, 10 ng/mL recombinant human IGF-1 (PeproTech, Inc. NJ, USA), 10 ng/mL recombinant human bFGF (Oriental Yeast Co., Ltd., Japan), 10 ng/mL recombinant human HGF (PeproTech, Inc.), and 0.2 mM 2-mercaptoethanol (2-ME, Nacalai tesque). The medium was then changed twice a week until day 38. Finally, on day 38, the medium was switched to Dulbecco’s Modified Eagle Medium (DMEM, Nacalai tesque, 08,488–55) supplemented with 2% horse serum (HS, Sigma-Aldrich, H1138), 2 mM L-glutamine (Nacalai tesque), 0.5% Penicillin–Streptomycin Mixed Solution, 5 μM SB, 10 ng/ml recombinant human IGF-1, and 0.1 mM 2-ME. The medium was changed three times per week until FCM analysis on day 82.

For Supplementary Fig. 1, cells were cultured on Matrigel or p421^64^. The other differentiation induction methods were the same as described above. FCM analysis was performed on day 74–84.

### RNA extraction and qRT-PCR

Total RNA was extracted with the ReliaPrep RNA Cell Miniprep system (Promega, WI, USA, Z6012). Complementary DNA was synthesized with ReverTra Ace qPCR RT Kit (TOYOBO Co., Ltd., Japan, FSQ-101). RT-qPCR was conducted with the Power SYBR Green PCR Master Mix (Applied Biosystems, MA, USA) and QuantStudio Real-Time PCR Systems (Thermo Fisher Scientific, Inc.). Technical duplicates were performed for each sample. The primers that were used are listed in Supplementary Table 1.

### ICC

For correlation analysis between myogenic protein expression on day 38 and MYF5 + % on day 82 (Fig. 2), cells were fixed in a culture plate with 2% paraformaldehyde (PFA, Nacalai tesque) for 10 min after being washed twice with Dulbecco’s Phosphate Buffered Saline (D-PBS, Nacalai tesque). Next, the cells were washed twice with D-PBS and blocked with Blocking One (Nacalai tesque) for 1 h. Following the blocking process, the cells were stained overnight at 4 °C with the MHC (eBioscience, Inc., CA, USA, 14–6503, 1:800) and MYOD1 (Abcam.plc, UK, ab133627, 1:500) antibodies diluted in D-PBS with 0.2% Triton X-100 (0.2% PBS-T, Sigma-Aldrich) and 10% Blocking One. After washing three times with 0.2% PBS-T, the cells were stained for 1 h at room temperature with the Alexa Fluor 568 conjugated goat-anti-mouse (Thermo Fisher Scientific, Inc., A11031, 1:500) and Alexa Fluor 488 conjugated goat-anti-rabbit (Thermo Fisher Scientific, Inc., A11034, 1:500) antibodies diluted in 0.2% PBS-T with 10% Blocking One and 1 μg/mL DAPI (Thermo Fisher Scientific, Inc.). Thereafter, the cells were washed two times with 0.2% PBS-T and three times with D-PBS.

For time-course observation of MHC expression (Supplementary Fig. 12), cells were fixed in a culture plate with 4% PFA for 15 min after being washed twice with D-PBS. Next, the cells were washed twice with D-PBS containing 0.1% Triton X-100 (0.1% PBS-T) and blocked with Blocking One for 1 h. Following the blocking process, the cells were stained overnight at 4 °C with the MHC (1:800) and MYOD1 (1:500) antibodies diluted in 0.1% PBS-T with 5% Blocking One. After washing twice with 0.1% PBS-T, the cells were stained for 1 h at room temperature with the Alexa Fluor 568 conjugated goat-anti-mouse (1:500) and Alexa Fluor 488 conjugated goat-anti-rabbit (1:500) antibodies diluted in 0.1% PBS-T with 5% Blocking One and 10 μg/mL Hoechst 33342 (Thermo Fisher Scientific, Inc.). Finally, the cells were washed two times with 0.1% PBS-T and three times with D-PBS.

The samples were observed with a BZ-X710 microscope (KEYENCE Corporation, Japan), and the MHC-positive area was calculated using the BZ-X analyzer software (KEYENCE Corporation) or ImageJ (Supplementary Fig. 1).

### FCM

The cells were dissociated for 30–60 min at 37 °C with DMEM supplemented with 2 mM L-glutamine, 0.5% Penicillin–Streptomycin Mixed Solution, 10% Dispase II (Godo Shusei Co., Ltd., Japan), 50 μg/mL Collagenase G (Meiji Seika Pharma Co., Ltd., Japan), and 10 μg/mL Collagenase H (Meiji Seika Pharma Co., Ltd.). After incubation, the cells were carefully detached via pipetting and the dissociation solution was removed by centrifugation. Thereafter, the cells were incubated in Accutase for 5 min at 37 °C to ensure only single cells were present. After filtration with a 40 μm cell strainer, followed by washing with Hanks’ Balanced Salt Solution with 1% BSA (HBSS, Thermo Fisher Scientific, Inc.), 1,000,000 cells were stained with 5 μg/mL Hoechst in HBSS and filtered with a 35 μm cell strainer. The cells were kept on ice until FCM analysis with BD LSRFortessa (Becton, Dickinson and Company, NJ, USA).

For Supplementary Fig. 1, the final differentiation induction efficiency was assessed using CDH13, a MuSC cell surface marker that we previously identified^46^. FCM was performed following our previously reported protocol^43^.

### Phase contrast cell imaging

The phase contrast cell images (1920 × 1440 pixels, 8-bit monochrome) were obtained with a BZ-X810 microscope (KEYENCE Corporation) using a 4 × objective lens. The same exposure time and gain value were used in all imaging. Twenty-one images per well were taken at different positions of the center part of the well at eight time points from day 14 to 38. We performed 34 wells of differentiation induction experiments and obtained a total of 5,712 images.

### Feature extraction

A FFT was applied to each cell image to obtain a FFT spectrum. Next, each component of the FFT spectrum was squared to obtain a FFT power spectrum, and shell integration was applied to the FFT power spectrum to obtain a rotationally symmetric 100-dimensional feature vector.

### Classification tasks

The overall framework of classification tasks is shown in Supplementary Fig. 10. The classifier was trained using the extracted feature vectors and labels of the images. We labeled each image based on the Task as follows; For Task 1, images with a MYF5 + % score of less than 10 were labeled as 1, and all other images were labeled as 0. For Task 2, images with a MYF5 + % score of more than 20 were labeled as 1, and all other images were labeled as 0. In each task, we constructed 50 independent classification models to evaluate accuracy fluctuations due to partitioning by cross validation. A four-fold cross-validation by dish was performed in each classification model. Utilizing the feature vector obtained from the FFT of each image, removing constant variables, PCA was performed to reduce multicollinearity effects. Furthermore, Partial Least Squares Discriminant Analysis (PLS-DA) was conducted using the principal components obtained from PCA and image labels. As a result, a linear combination of the 10-dimensional principal components from PCA and the 1-dimensional principal component obtained from PLS was used as explanatory variables for the random forest classification model. The performance of the trained classifier was assessed by measuring predictive ability on test data not included in the training set, obtaining the average evaluation metrics in validation. Source code is available in (https://github.com/TakuTsuzuki/MuSCs_classifier).

### Statistical analysis

For correlation analysis in Fig. 2 and Supplementary Fig. 1, a test for no correlation was performed. The statistical significance of Fig. 3 and Fig. 4 was determined using a Mann–Whitney U test. These results were corrected for multiple comparisons using the Benjamini–Hochberg correction.

## Supplementary Information

Supplementary Information.

## Data Availability

Source code that supports the results is available in (https://github.com/TakuTsuzuki/MuSCs_classifier). The data will be made available by the corresponding author upon reasonable request.
