# Supplementary material for: Early and non-destructive prediction of the differentiation efficiency of human induced pluripotent stem cells using imaging and machine learning
Source: Sci Rep. 2025 Jul 23;15:26821. doi: 10.1038/s41598-025-11108-5 (PMC12287433; doi:10.1038/s41598-025-11108-5)
Supplement: Supplementary file 1 — Supplementary Information. [file 41598_2025_11108_MOESM1_ESM.pdf]

Supplementary Information

Early and non-destructive prediction of the differentiation efficiency of human induced pluripotent stem cells using imaging and machine learning

Miki Arai Hojo, Taku Tsuzuki, Yosuke Ozawa, Toshiyuki Araki, and Hidetoshi Sakurai

Supplementary Figure 1

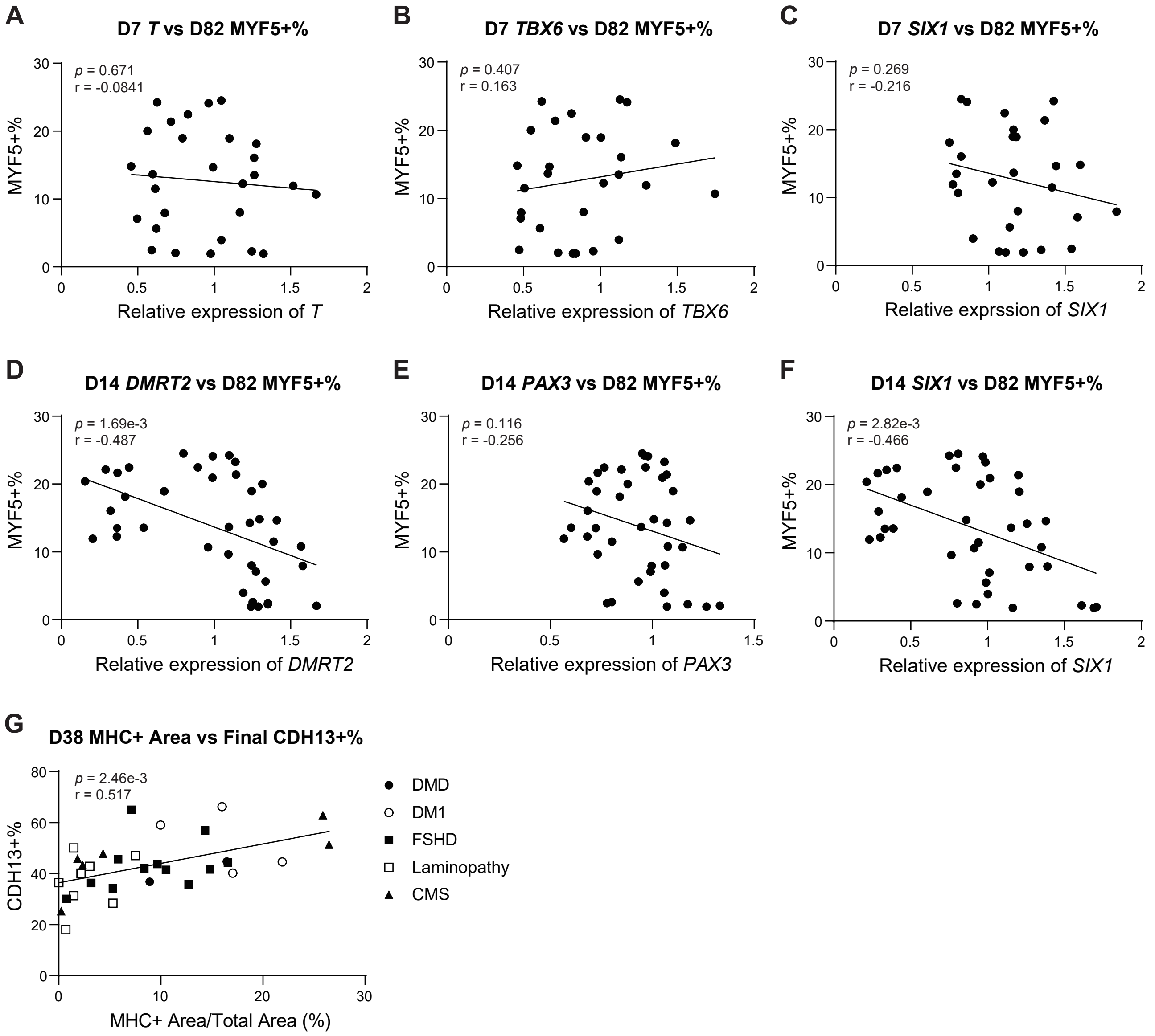

Supplementary Figure 1: The dermomyotome induction phase is not suitable for the prediction of MuSC induction efficiency on day 82

(A-C) Correlation analysis between the MYF5+% score on day 82 and relative mRNA expression of *T* (A), *TBX6* (B), or *SIX1* (C) on day 7 (normalized to *HPRT1*). Data are pooled from 28 wells of experiments. The mRNA expression levels were examined in separate wells from those used for MYF5+% analysis and were cultured using the same experimental conditions.

(D-F) Correlation analysis between the MYF5+% score on day 82 and relative mRNA expression of *DMRT2* (D), *PAX3* (E), or *SIX1* (F) on day 14 (normalized to *HPRT1*). Data are pooled from 39 wells of experiments. The mRNA expression levels were examined using separate wells from those used for MYF5+% analysis and were cultured using the same experimental conditions.

(G) Correlation analysis between the CDH13+% score on day 74–84 and MHC+ area calculated from images of ICC on day 38. Data are pooled from 32 wells across five patient-derived iPSC clones: Duchenne muscular dystrophy (DMD), myotonic dystrophy type 1 (DM1), facioscapulohumeral muscular dystrophy (FSHD) type 1, laminopathy, congenital myasthenic syndrome (CMS) with a *GFPT1* mutation. Five images per well were taken at different positions.

Supplementary Figure 2

A

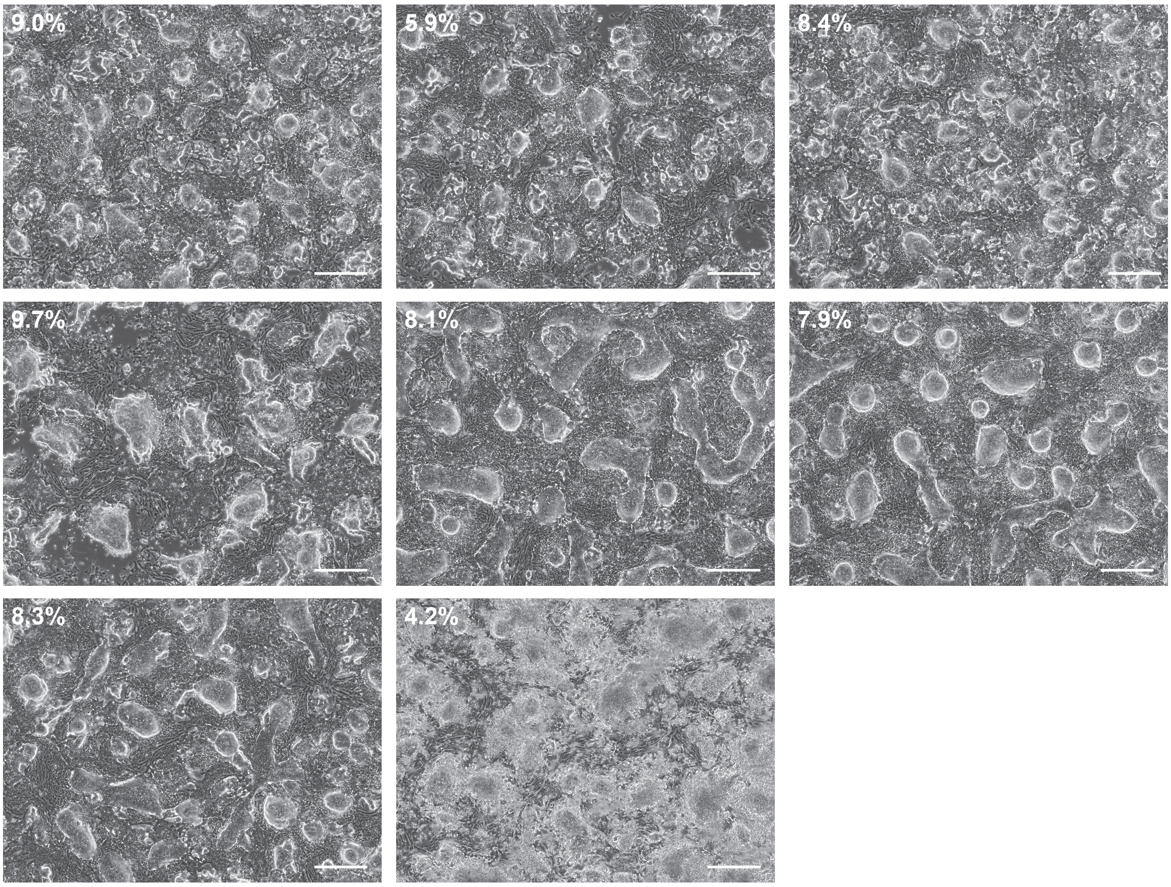

B

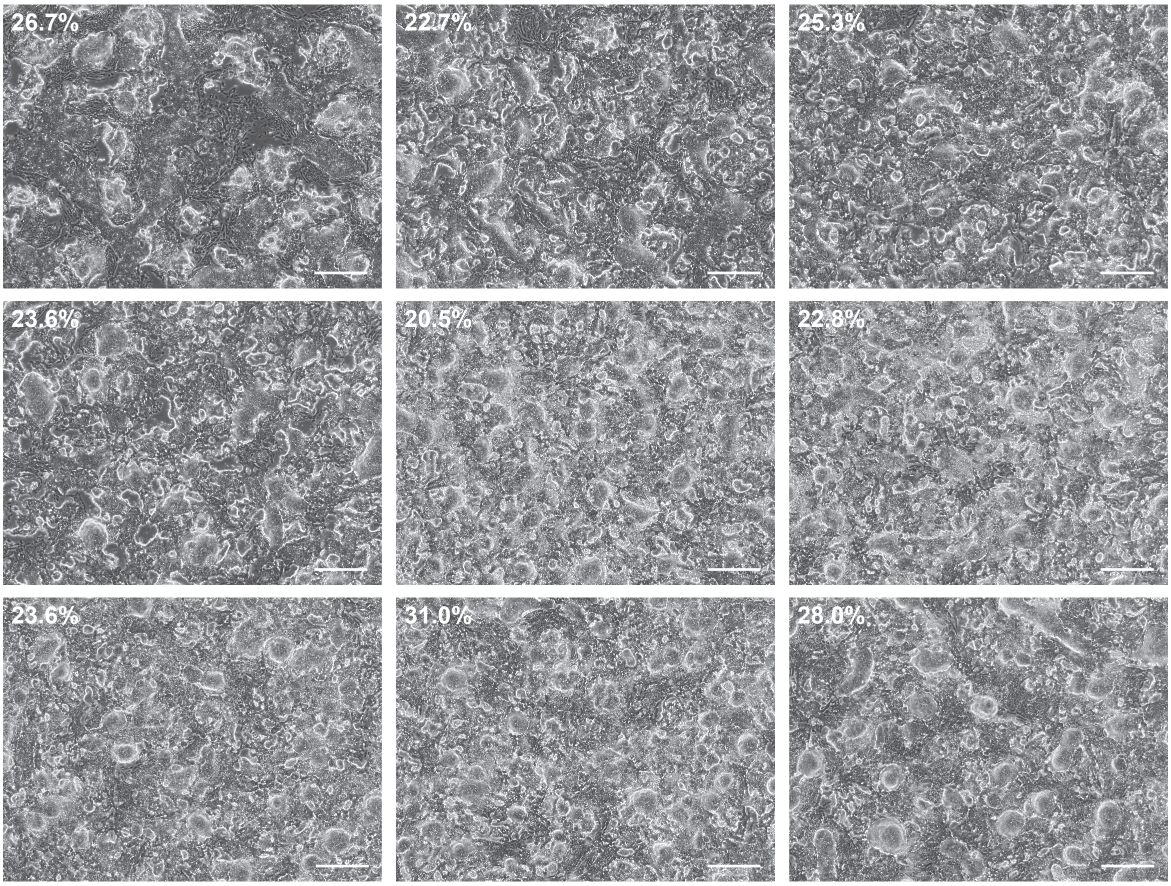

Supplementary Figure 2: Phase contrast cell images on day 14

(A) Images from all samples that had a MYF5+% score of less than 10 on day 82. Images are representative of 21 points of view per well. The scale bar represents 500 μm.

(B) Images from all samples that had a MYF5+% score of more than 20 on day 82. Images are representative of 21 points of view per well. The scale bar represents 500 μm.

Supplementary Figure 3

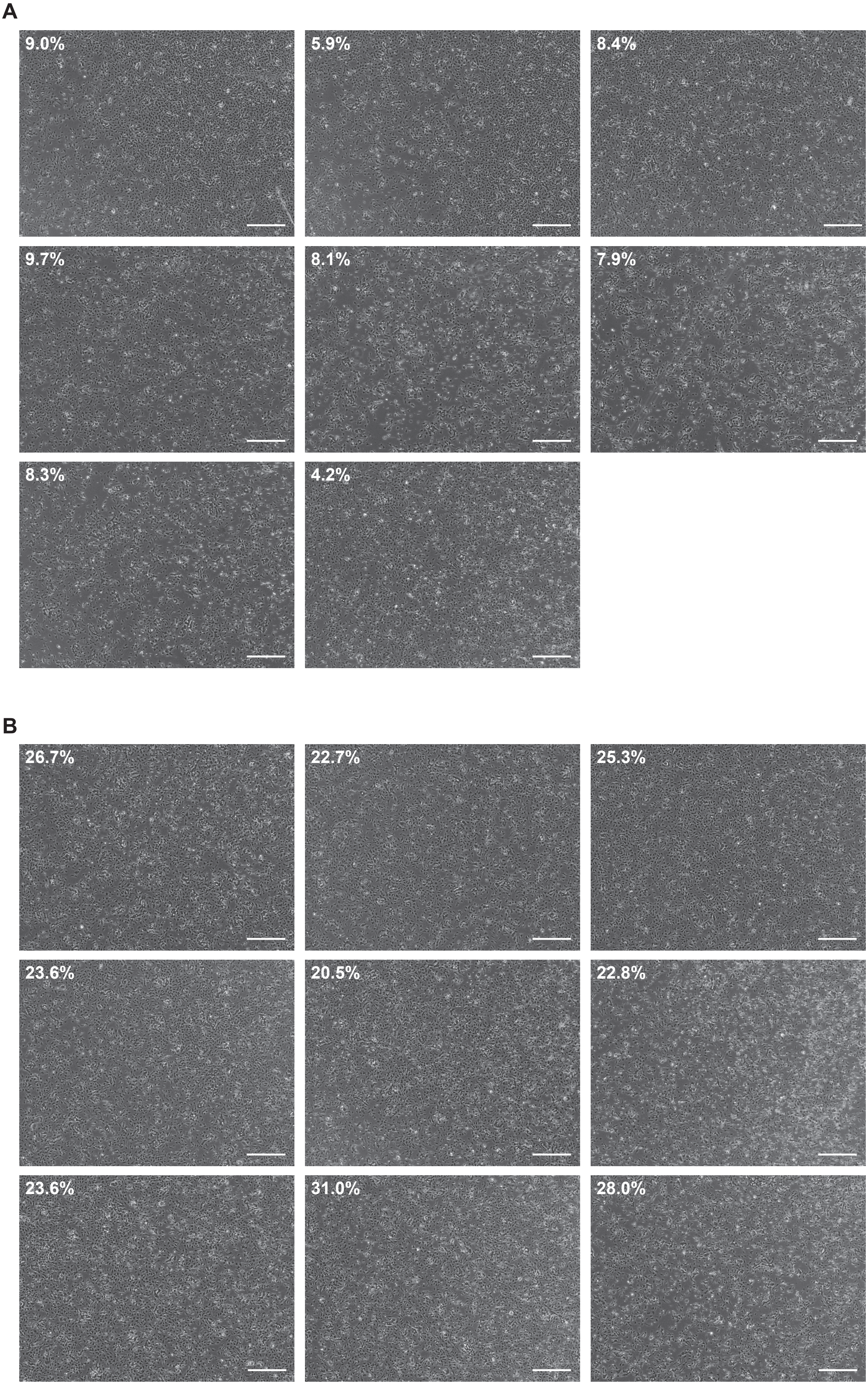

Supplementary Figure 3: Phase contrast cell images on day 17

(A) Images from all samples that had a MYF5+% score of less than 10 on day 82. Images are representative of 21 points of view per well. The scale bar represents 500 μm.

(B) Images from all samples that had a MYF5+% score of more than 20 on day 82. Images are representative of 21 points of view per well. The scale bar represents 500 μm.

Supplementary Figure 4

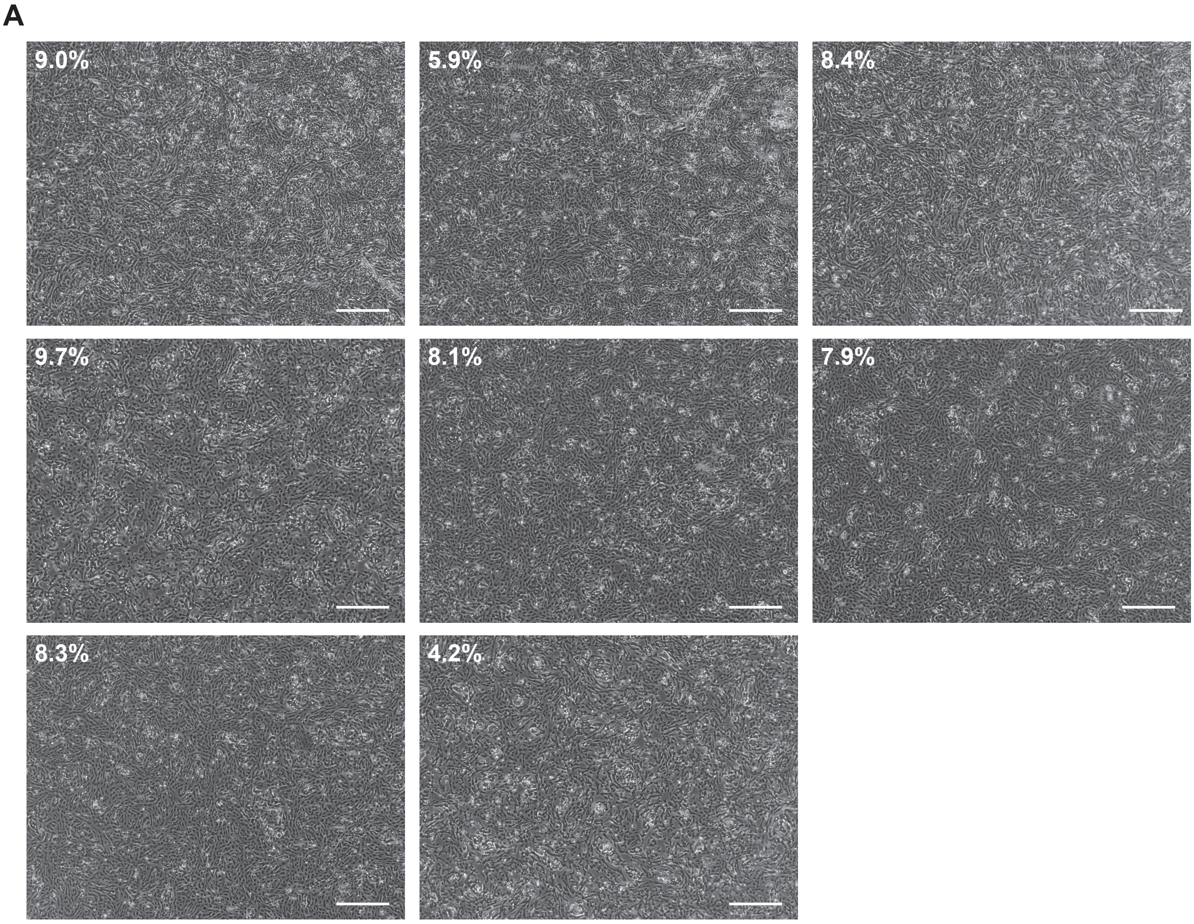

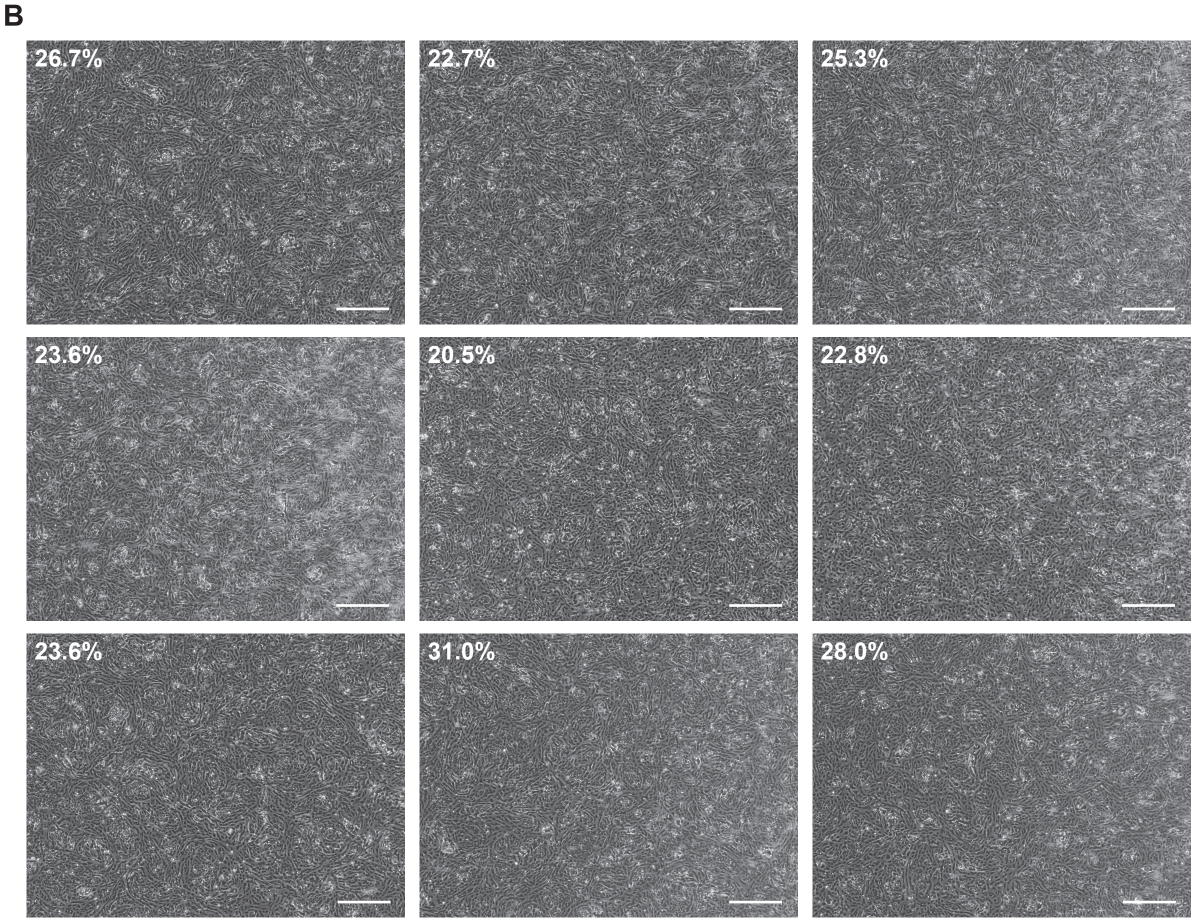

Supplementary Figure 4: Phase contrast cell images on day 20

(A) Images from all samples that had a MYF5+% score of less than 10 on day 82. Images are representative of 21 points of view per well. The scale bar represents 500 μm.

(B) Images from all samples that had a MYF5+% score of more than 20 on day 82. Images are representative of 21 points of view per well. The scale bar represents 500 μm.

Supplementary Figure 5

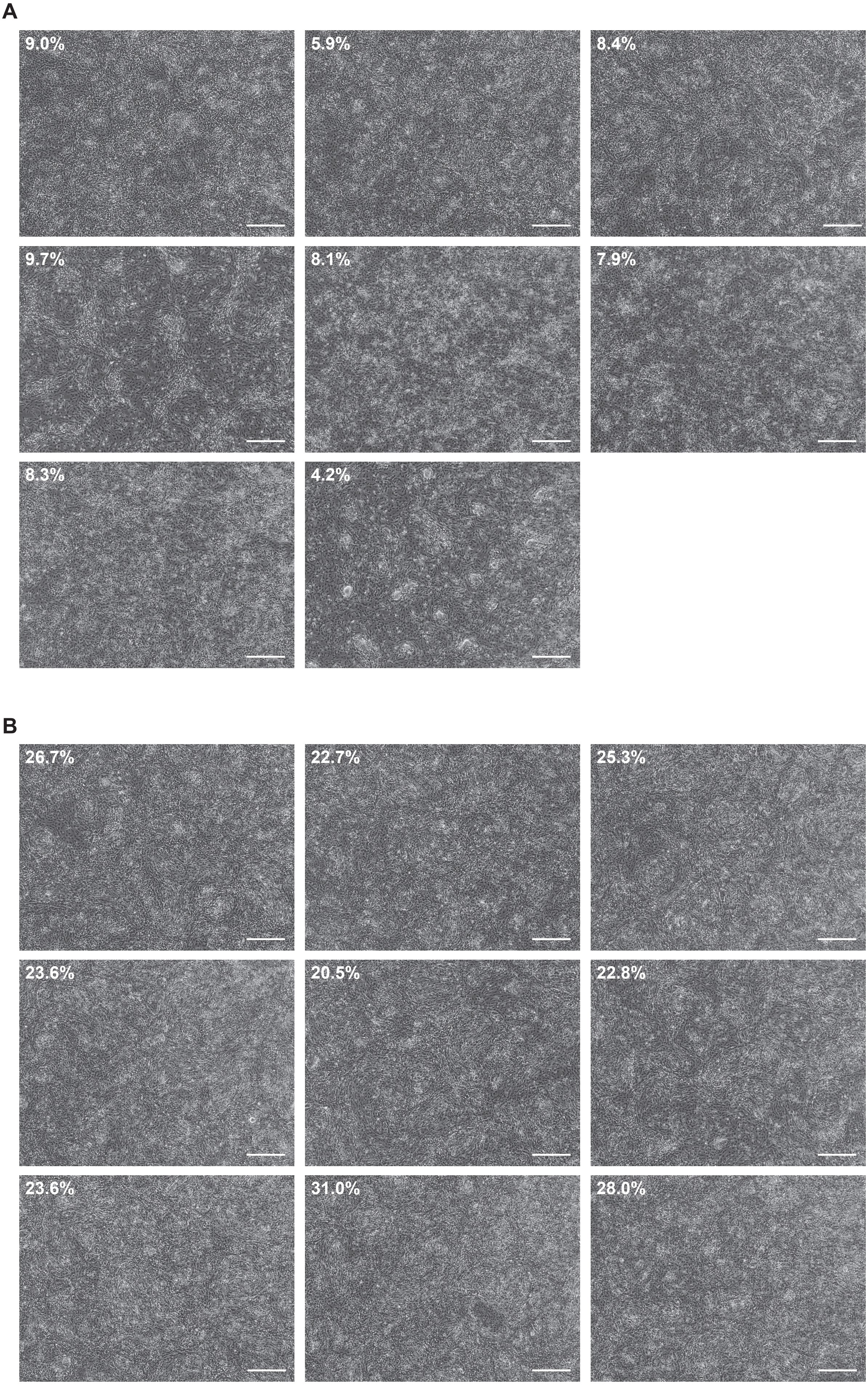

Supplementary Figure 5: Phase contrast cell images on day 24

(A) Images from all samples that had a MYF5+% score of less than 10 on day 82. Images are representative of 21 points of view per well. The scale bar represents 500 μm.

(B) Images from all samples that had a MYF5+% score of more than 20 on day 82. Images are representative of 21 points of view per well. The scale bar represents 500 μm.

Supplementary Figure 6

A

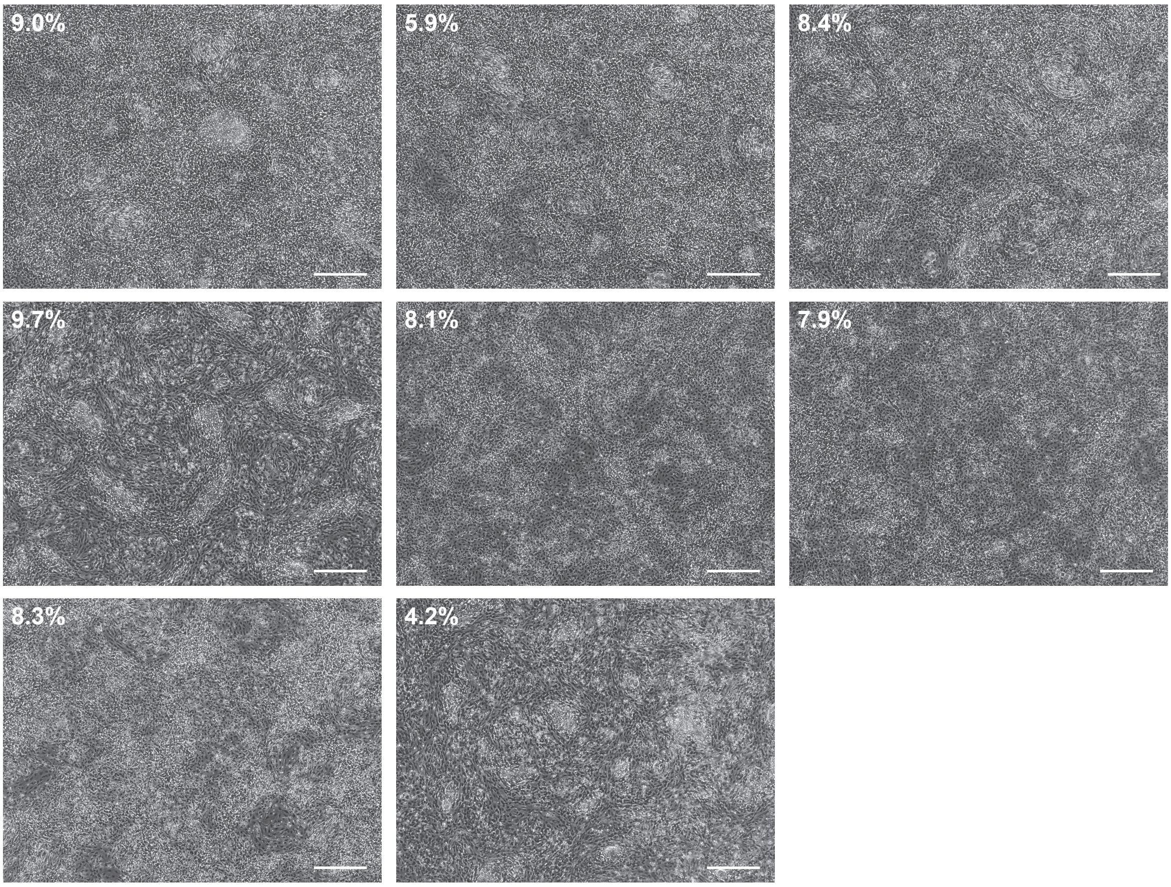

B

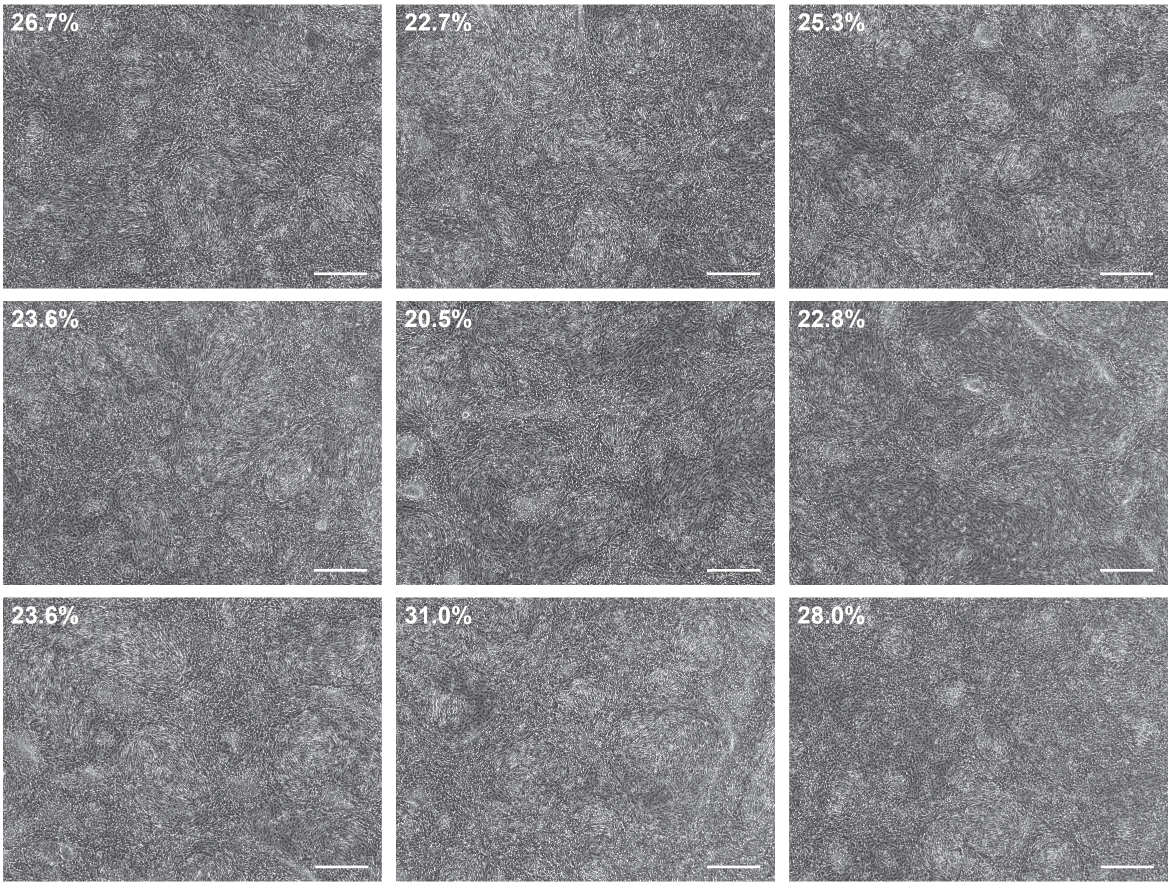

Supplementary Figure 6: Phase contrast cell images on day 27

(A) Images from all samples that had a MYF5+% score of less than 10 on day 82. Images are representative of 21 points of view per well. The scale bar represents 500 μm.

(B) Images from all samples that had a MYF5+% score of more than 20 on day 82. Images are representative of 21 points of view per well. The scale bar represents 500 μm.

Supplementary Figure 7

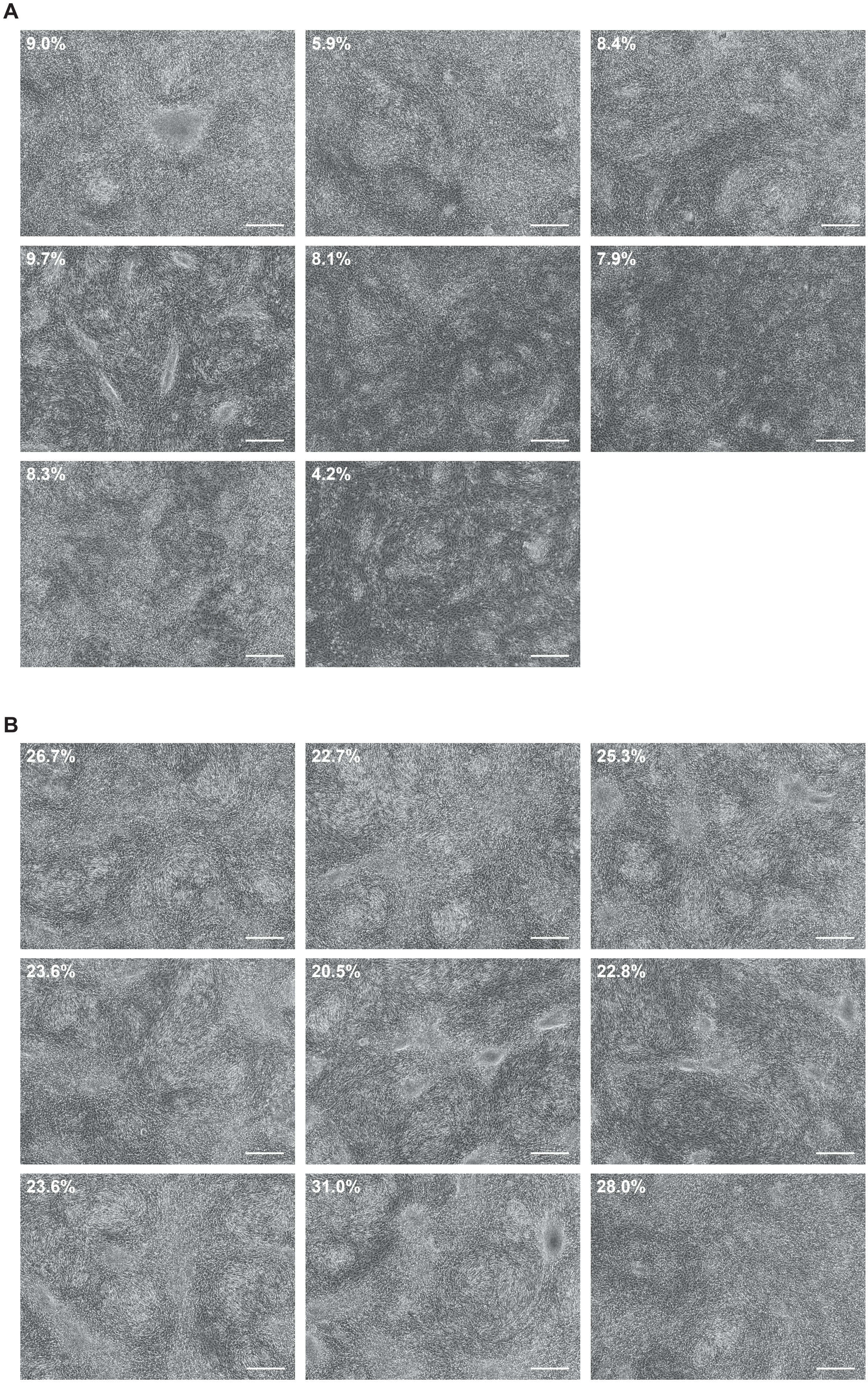

Supplementary Figure 7: Phase contrast cell images on day 31

(A) Images from all samples that had a MYF5+% score of less than 10 on day 82. Images are representative of 21 points of view per well. The scale bar represents 500 μm.

(B) Images from all samples that had a MYF5+% score of more than 20 on day 82. Images are representative of 21 points of view per well. The scale bar represents 500 μm.

Supplementary Figure 8

A

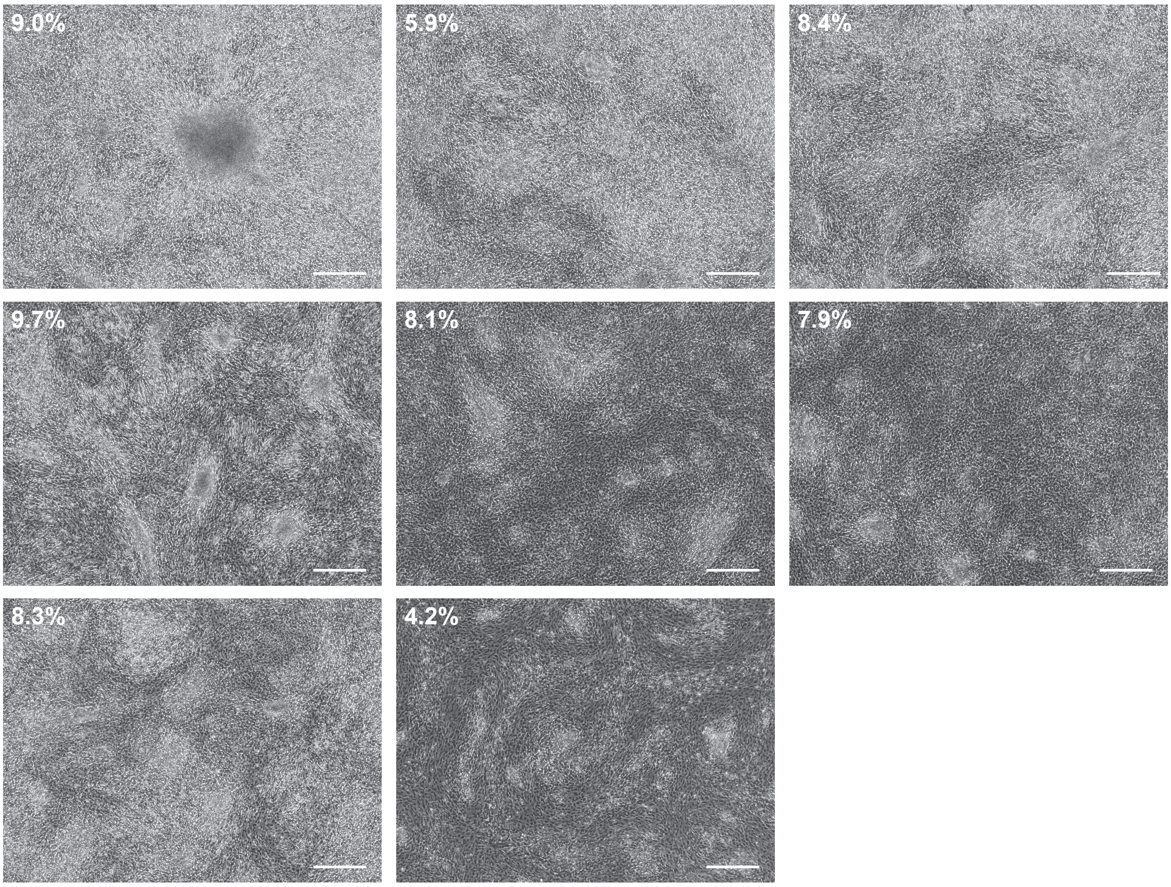

B

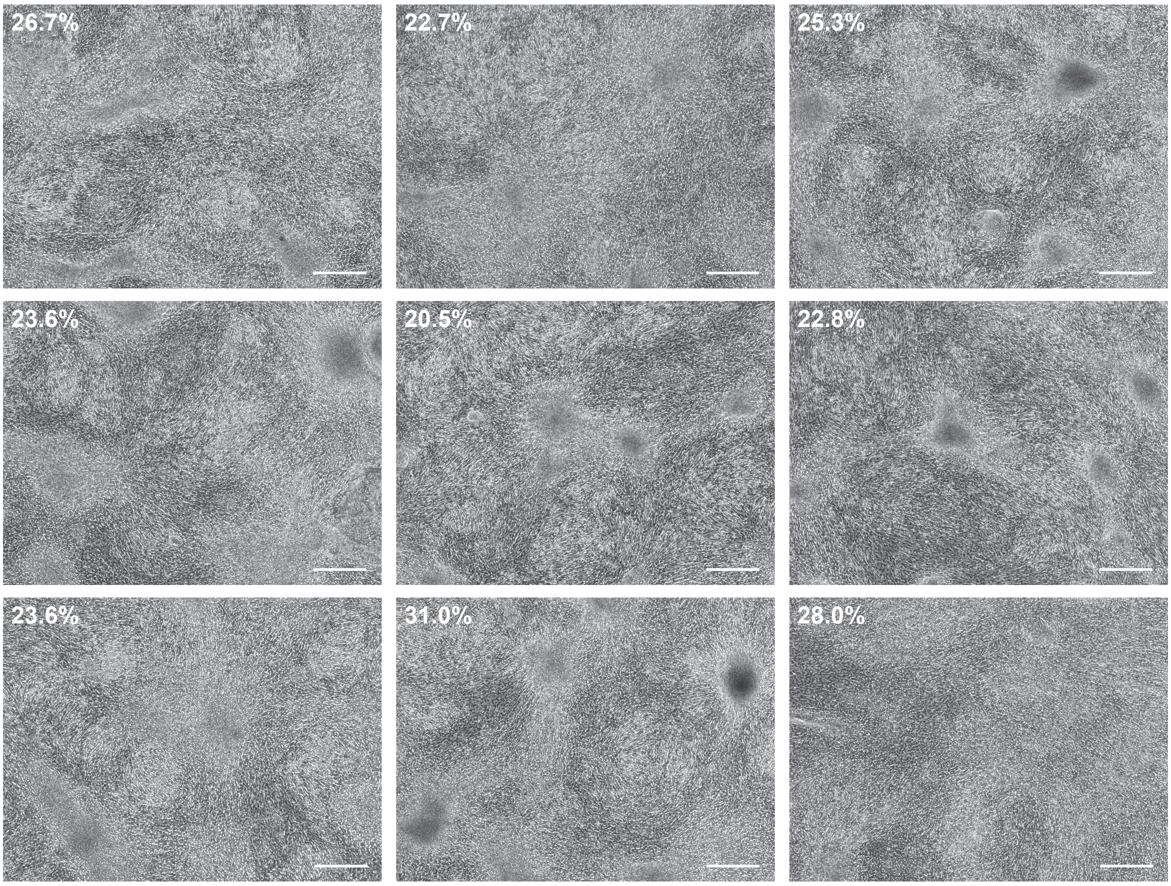

Supplementary Figure 8: Phase contrast cell images on day 34

(A) Images from all samples that had a MYF5+% score of less than 10 on day 82. Images are representative of 21 points of view per well. The scale bar represents 500 μm.

(B) Images from all samples that had a MYF5+% score of more than 20 on day 82. Images are representative of 21 points of view per well. The scale bar represents 500 μm.

Supplementary Figure 9

A

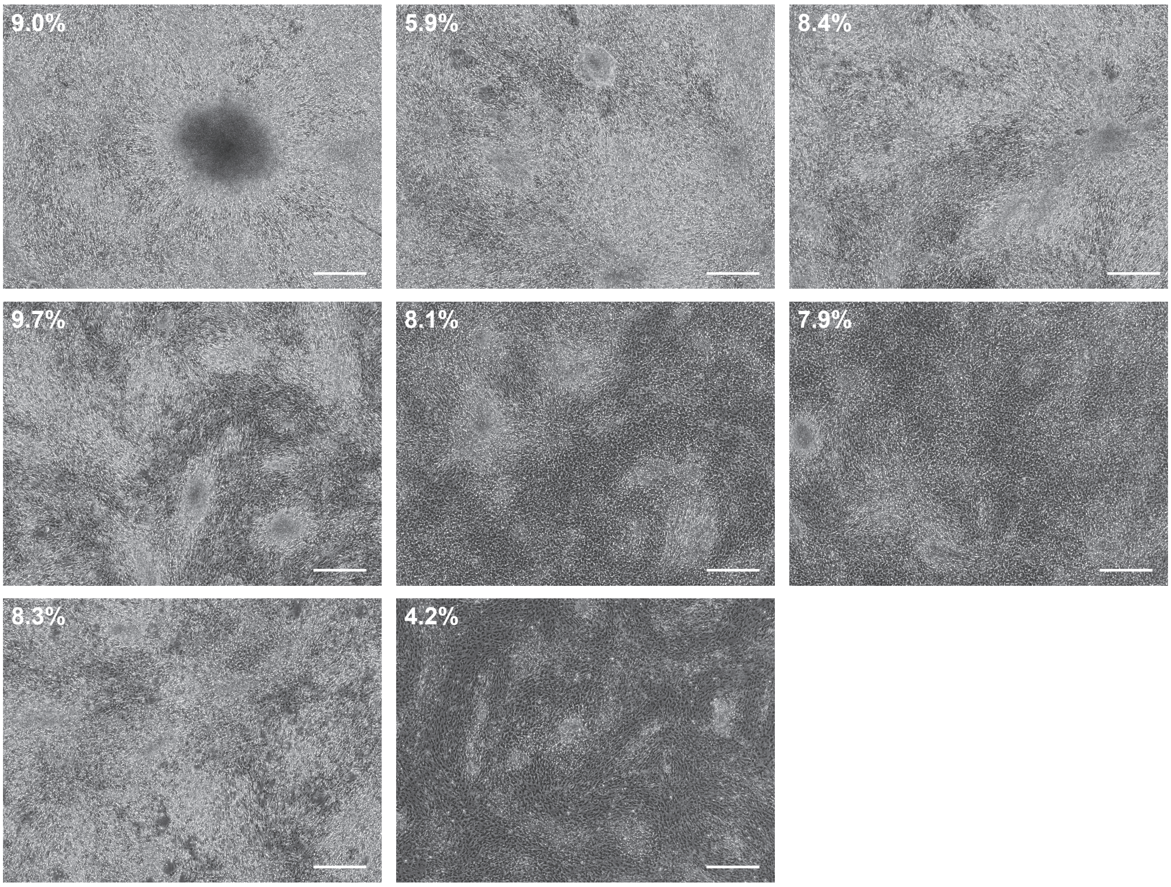

B

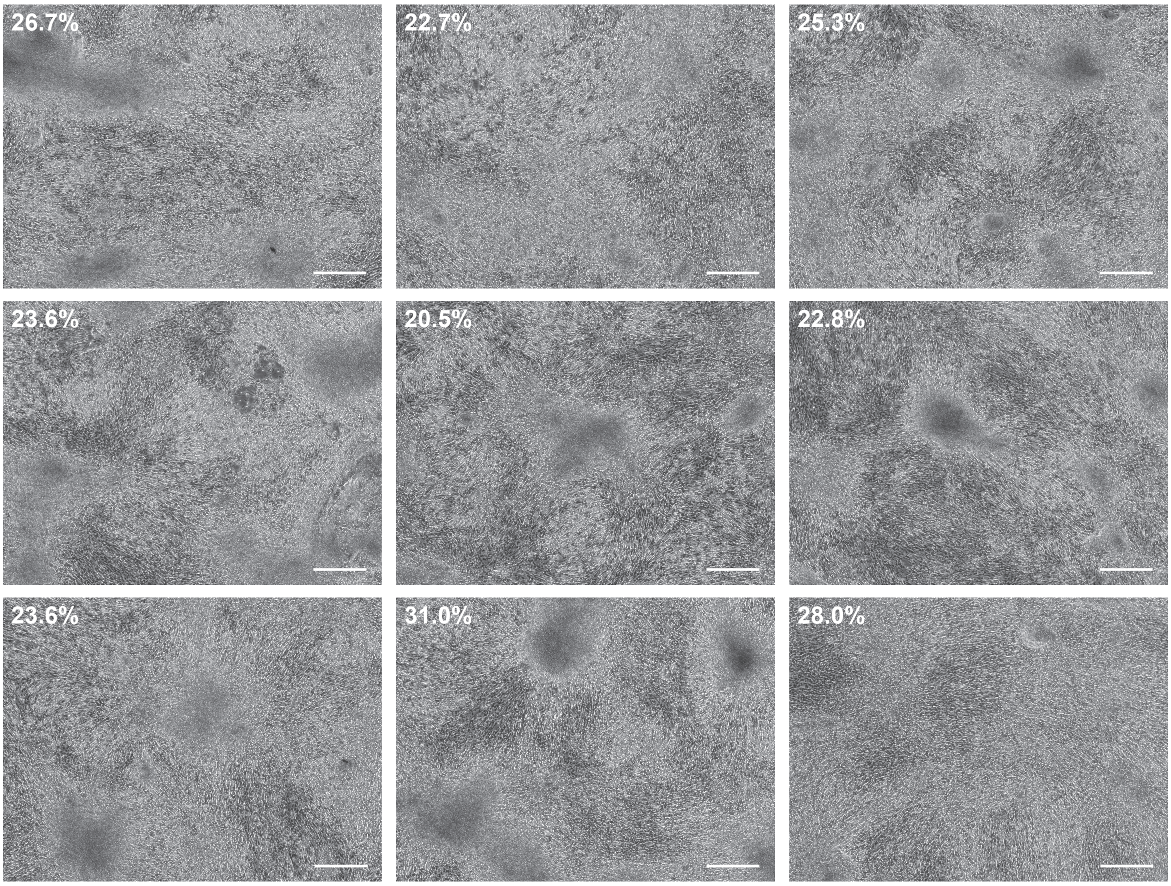

Supplementary Figure 9: Phase contrast cell images on day 38

(A) Images from all samples that had a MYF5+% score of less than 10 on day 82. Images are representative of 21 points of view per well. The scale bar represents 500 μm.

(B) Images from all samples that had a MYF5+% score of more than 20 on day 82. Images are representative of 21 points of view per well. The scale bar represents 500 μm.

Supplementary Figure 10

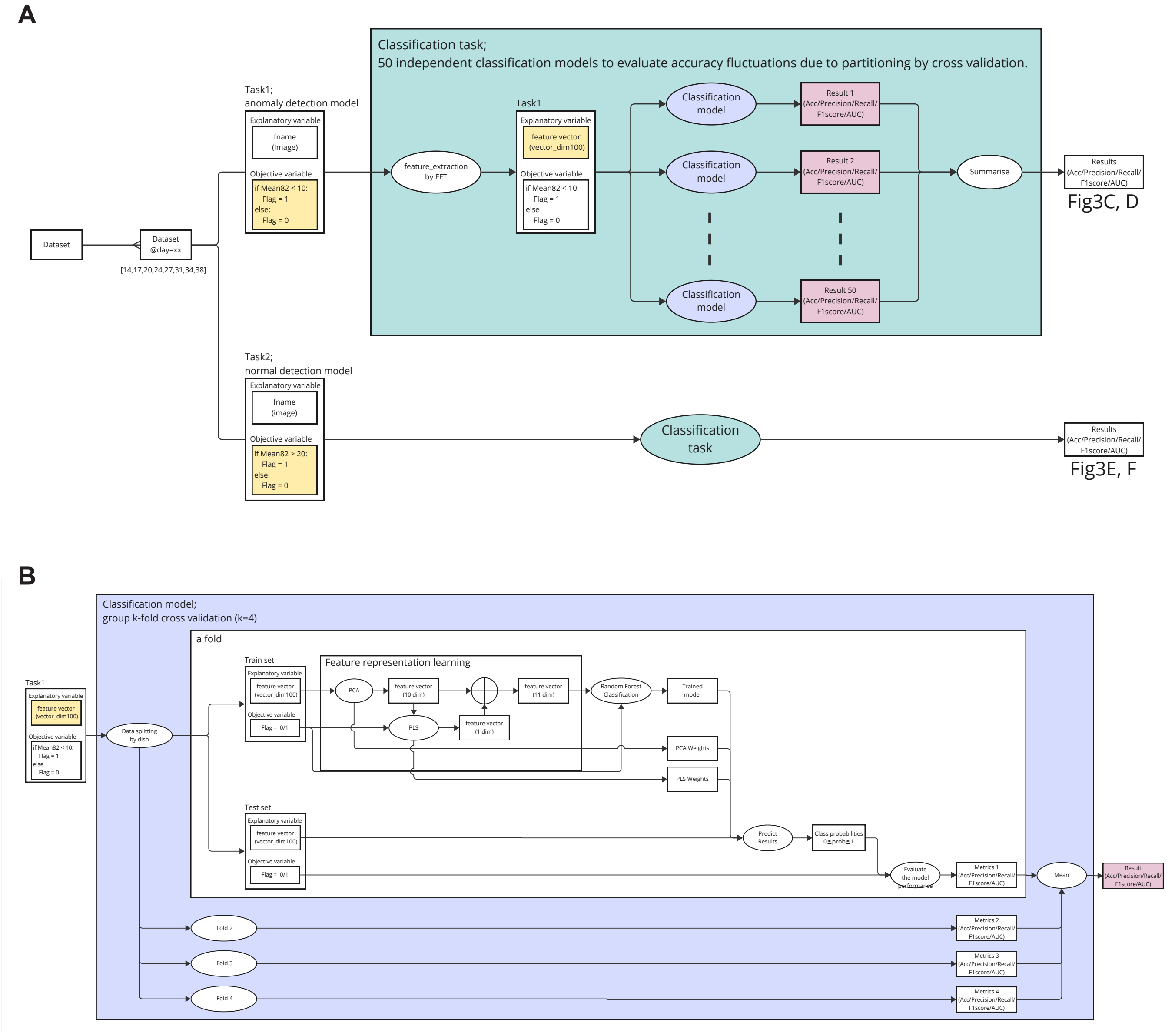

Supplementary Figure 10: Overview of the classification model architecture and its performance evaluation process

(A) Schematic explanation of the overall classification task implementation. The green box shows the process of taking the Task 1 dataset as input and obtaining the evaluation results (Figure 3).

(B) Schematic explanation of the architecture of the classification model and its performance evaluation using Cross validation.

Supplementary Figure 11

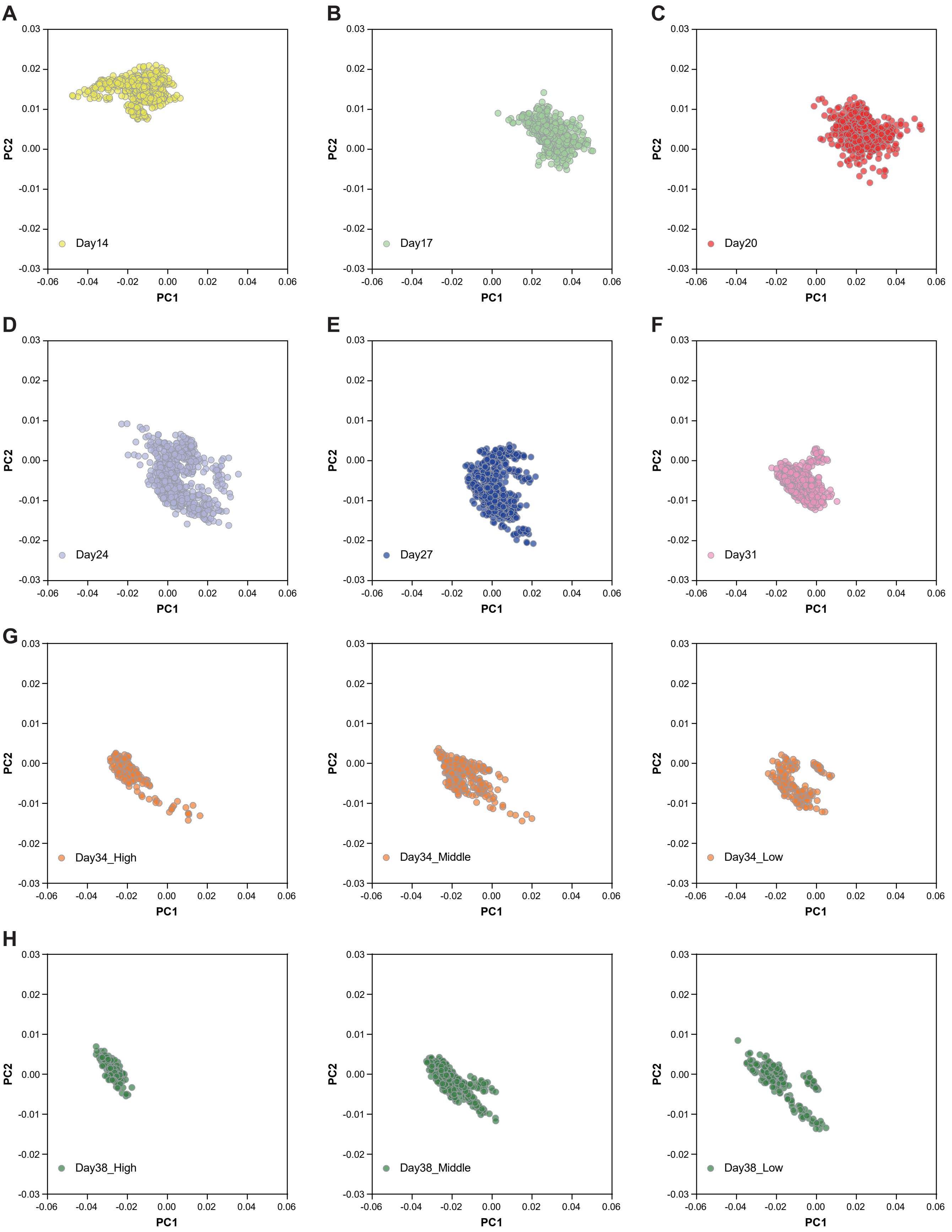

Supplementary Figure 11: FFT could capture the morphological change in cells throughout dermomyotome to myogenic differentiation

(A-H) PCA for feature vectors from images on days 14 (A), 17 (B), 20 (C), 24 (D), 27 (E), 31 (F), 34 (G), and 38 (H) (High: MYF5+% > 20%, Middle: 10–20%, Low: <10%).

Supplementary Figure 12

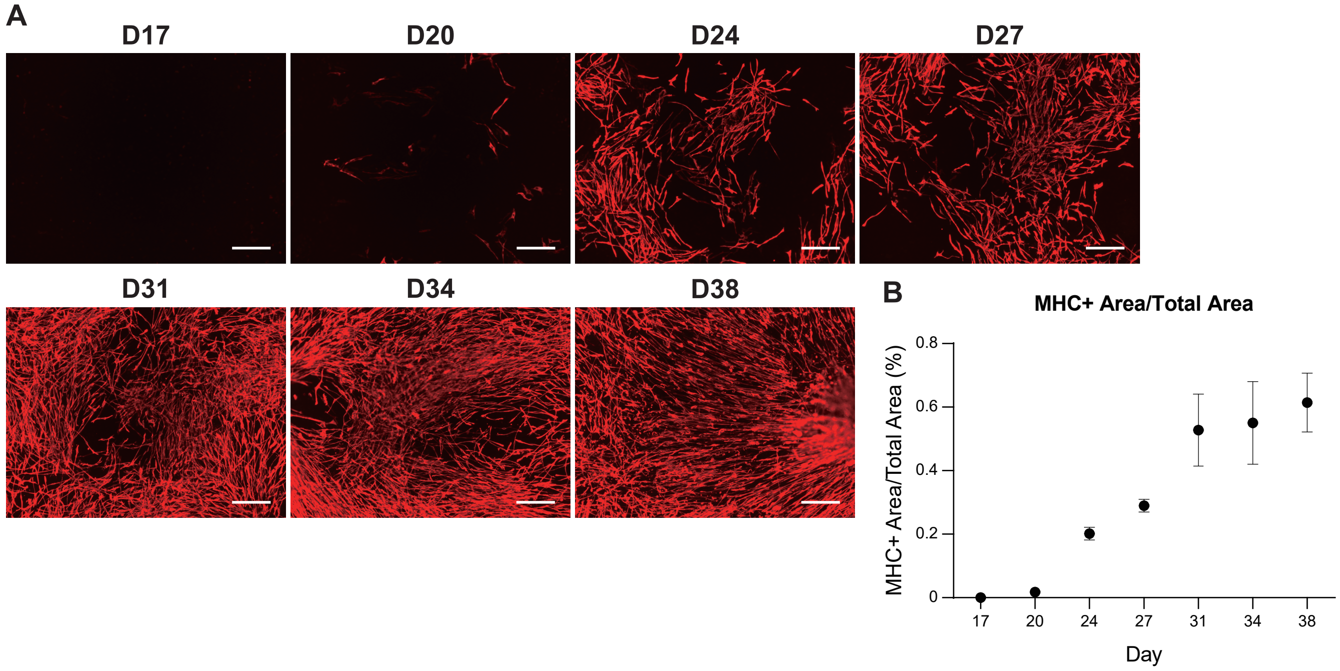

Supplementary Figure 12: The possibility that the classifier also used morphological change related to MHC expression for prediction.

(A) The results of ICC for MHC from day 17 to 38. The scale bar represents 500 μm.

(B) MHC+ area calculated using images of ICC between day 17 and 38. Four images per well were obtained at different positions of the well.

Supplementary Table 1 Primer List of qRT-PCR

Gene	Forward Sequence	Reverse Sequence
HPRT1	TGGCGTCGTGATTAGTGATG	ATCTCGAGCAAGACGTTCAG
T	ACCCAGTTCATAGCGGTGAC	CATTGGGAGTACCCAGGTTG
TBX6	AGCCTGTGTCTTTCCATCGT	AGGCTGTCACGGAGATGAAT
SIX1	AGTTCTCGCCTCACAACCAC	ACACCCCTCGACTTCTCCTT
DMRT2	GAACCACCAAGCAAGGACTTC	CCCAGACCCTGAATACTGCAT
PAX3	AGGAAGGAGGCAGAGGAAAG	CAGCTGTTCTGCTGTGAAGG
MYH3	GCAGATTGAGCTGGAAAAGG	TCAGCTGCTCGATCTCTTCA
MYOD1	ACGTGAGGACGAGCATGTG	GTGCAGCGCTTGAGTGTCT
MYOG	TGGGCGTGTAAGGTGTGTAA	CGATGTACTGGATGGCACTG
